# Supplementary material for: RZZ‐Spindly and CENP‐E form an integrated platform to recruit dynein to the kinetochore corona
Source: EMBO J. 2023 Nov 20;42(24):e114838. doi: 10.15252/embj.2023114838 (PMC10711656; doi:10.15252/embj.2023114838)
Supplement: Supplementary file 1 — Appendix [file EMBJ-42-e114838-s004.pdf]

Appendix

RZZ-Spindly and CENP-E form an integrated platform to recruit Dynein to the kinetochore corona

By Verena Cmentowski et al.

Including Appendix Figures S1 to S4 and their legends

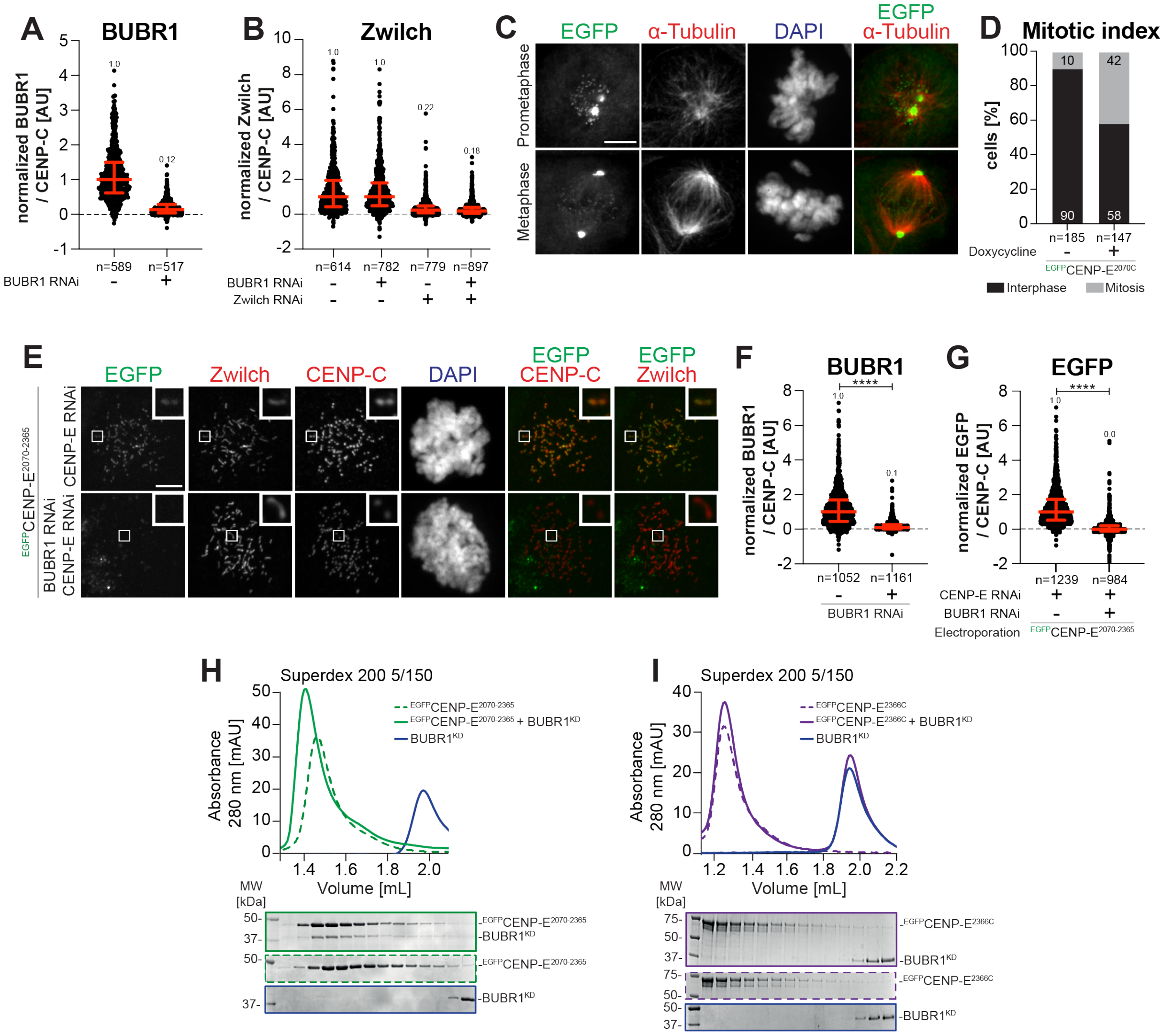

Appendix Figure S1

(**A-B**) Quantification of BUBR1 and Zwilch levels of cells treated as shown in Figure 1C-D. n refers to individually measured kinetochores. Red bars represent median and interquartile range. (**C**) Representative images showing the localization of ^{EGFP}CENP-E^{2070C} in prometaphase and metaphase stable DLD-1 cells. 32 h after cells were seeded, protein expression was induced through addition of 300 ng/mL doxycycline for 16 h before fixation. CENP-C was used to visualize kinetochores and DAPI to stain DNA. Scale bar: 5 µm. (**D**) Mitotic index analysis of a stable DLD-1 cell line expressing ^{EGFP}CENP-E^{2070C}. 32 h after seeding, cells were treated with 300 ng/mL doxycycline or DMSO for 16 h before fixation. n refers to the number of analyzed cells. (**E**) Representative images showing the localization of ^{EGFP}CENP-E$^{2070\text{-}2365}$ in prometaphase in presence or absence of BUBR1. BUBR1 RNAi treatment was performed with 100 nM siRNA and CENP-E RNAi treatment with 60 nM siRNA. 13 h after CENP-E and BUBR1 RNAi treatment HeLa cells were electroporated with recombinant ^{EGFP}CENP-E$^{2070\text{-}2365}$. Following an 8 h recovery, cells were synchronized in G2 phase with 9 µM RO3306 for 15 h and then released into mitosis. Subsequently, cells were immediately treated with 3.3 µM Nocodazole for an additional hour. CENP-C was used to visualize kinetochores and DAPI to stain DNA. Scale bar: 5 µm. (**F-G**) Quantification of BUBR1 and EGFP levels at kinetochores of the experiment shown in panel E. n refers to individually measured kinetochores. Statistical analysis was performed with a nonparametric t test comparing two unpaired groups (Mann–Whitney test). Symbols indicate: n.s. = $p > 0.05$, * = $p \leq 0.05$, ** = $p \leq 0.01$, *** = $p \leq 0.001$, **** = $p \leq 0.0001$. Red bars represent median and interquartile range. (**H-I**) Analytical SEC binding assays between the BUBR1 kinase domain (KD) and different ^{EGFP}CENP-E constructs. The profile of the complex is represented as a continuous line, and the individual CENP-E constructs with a dashed line. BUBR1: 8 µM, CENP-E constructs: 16 µM.

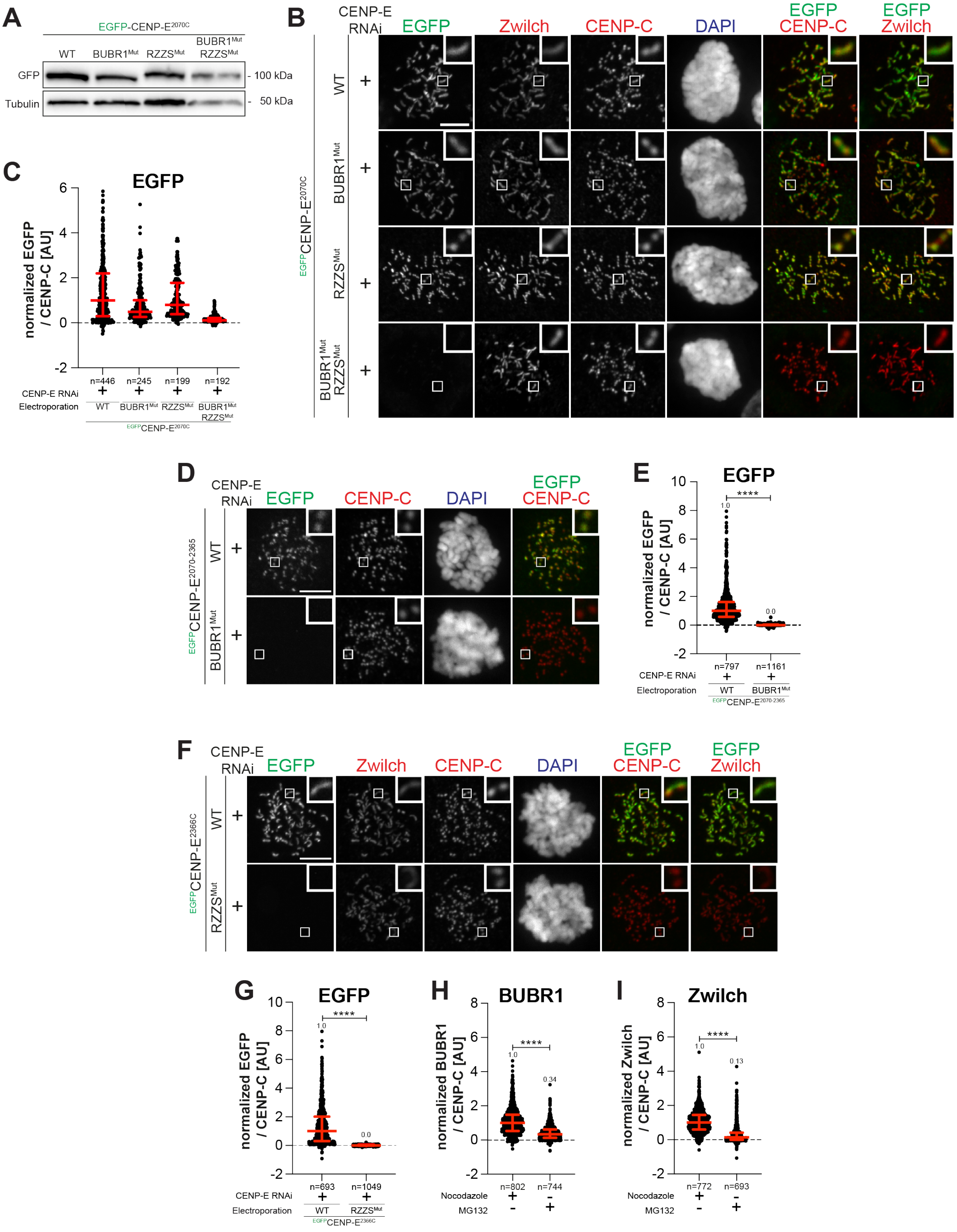

Appendix Figure S2

(**A**) Immunoblot of mitotic DLD-1 cells stably expressing different ^{EGFP}CENP-E$^{2070\mathrm{C}}$ constructs, treated as shown in (Figure 2D) and probed with the indicated antibodies. 50 µg of cleared lysate was used for each

condition, and Tubulin is shown as a loading control. (**B**) Representative images showing the localization of different ^{EGFP}CENP-E^{2070C} constructs in prometaphase after depletion of CENP-E with 60 nM siRNA. 13 h after RNAi treatment HeLa cells were electroporated with recombinant ^{EGFP}CENP-E^{2070C} constructs as indicated. Following an 8 h recovery, cells were synchronized in G2 phase with 9 µM RO3306 for 15 h and then released into mitosis. Subsequently, cells were immediately treated with 3.3 µM Nocodazole for an additional hour. CENP-C was used to visualize kinetochores and DAPI to stain DNA. Scale bar: 5 µm. (**C**) Quantification of EGFP levels at kinetochores of the experiment shown in panel B. n refers to individually measured kinetochores. Red bars represent median and interquartile range. (**D**) Representative images showing the localization of different ^{EGFP}CENP-E$^{2070-2365}$ constructs in prometaphase after depletion of CENP-E with 60 nM siRNA. 13 h after RNAi treatment HeLa cells were electroporated with recombinant ^{EGFP}CENP-E$^{2070-2365}$ constructs as indicated. Following an 8 h recovery, cells were synchronized in G2 phase with 9 µM RO3306 for 15 h and then released into mitosis. Subsequently, cells were immediately treated with 3.3 µM Nocodazole for an additional hour. CENP-C was used to visualize kinetochores and DAPI to stain DNA. Scale bar: 5 µm. (**E**) Quantification of EGFP levels at kinetochores of the experiment shown in panel D. n refers to individually measured kinetochores. Statistical analysis (also for H-I) was performed with a nonparametric t test comparing two unpaired groups (Mann–Whitney test). Symbols indicate: n.s. = $p > 0.05$, * = $p \leq 0.05$, ** = $p \leq 0.01$, *** = $p \leq 0.001$, **** = $p \leq 0.0001$. Red bars represent median and interquartile range. (**F**) Representative images showing the localization of different ^{EGFP}CENP-E^{2366C} constructs in prometaphase after depletion of CENP-E with 60 nM siRNA. 13 h after RNAi treatment HeLa cells were electroporated with recombinant ^{EGFP}CENP-E^{2366C} constructs as indicated. Following an 8 h recovery, cells were synchronized in G2 phase with 9 µM RO3306 for 15 h and then released into mitosis. Subsequently, cells were immediately treated with 3.3 µM Nocodazole for an additional hour. CENP-C was used to visualize kinetochores and DAPI to stain DNA. Scale bar: 5 µm. This experiment is part of a larger experiment where we also compared DMSO-treated with Reversine-treated cells. Both samples shown here were treated with DMSO (see Methods). The WT control row is also displayed in Figure EV2E. (**G**) Quantification of EGFP levels at kinetochores of the experiment shown in panel F. n refers to individually measured kinetochores. Red bars represent median and interquartile range. (**H-I**) Comparison of BUBR1 and Zwilch levels at kinetochores in Nocodazole and MG132 arrested cells. n refers to individually measured kinetochores.

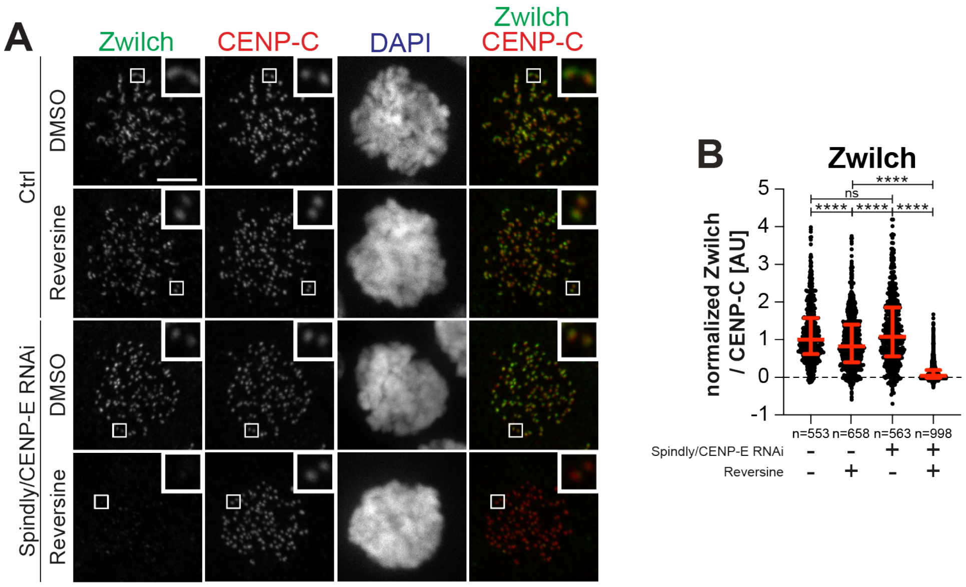

Appendix Figure S3

(**A**) Representative images showing kinetochore levels of Zwilch after depletion of Spindly and CENP-E. Spindly RNAi treatment was performed with 50 nM siRNA. 24 h after Spindly RNAi treatment, HeLa cells were transfected with 60 nM CENP-E siRNA. 8 h after transfection, cells were synchronized in G2 phase with 9 µM RO3306 for 15 h and then released into mitosis. Subsequently, cells were immediately treated with 3.3 µM Nocodazole, 10 µM MG132 and, where indicated, with 500 nM Reversine, for an additional hour. CENP-C was used to visualize kinetochores and DAPI to stain DNA. Scale bar: 5 µm. (**B**) Quantification of Zwilch levels at kinetochores of the experiment shown in panel A. n refers to individually measured kinetochores. Statistical analysis was performed with a nonparametric t test comparing two unpaired groups (Mann–Whitney test). Symbols indicate: n.s. = $p > 0.05$, * = $p \leq 0.05$, ** = $p \leq 0.01$, *** = $p \leq 0.001$, **** = $p \leq 0.0001$. Red bars represent median and interquartile range.

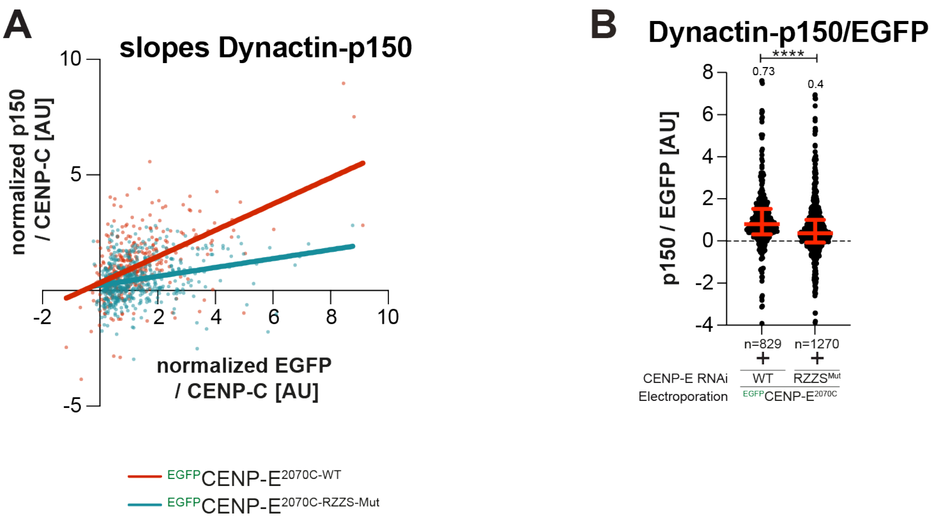

Appendix Figure S4

(**A**) Linear fitting through the distribution of data points reporting for each kinetochore the CENP-C–normalized EGFP-CENP-E intensity on the x-axis and the CENP-C-normalized Dynactin-p150 intensity on the y-axis from the experiment shown in Figure 5A-D. (**B**) Scatter dot plot of the ratio of CENP-C–normalized Dynactin-p150 intensity over CENP-C–normalized EGFP-CENP-E intensity from the experiment shown in Figure 5A-D. n refers to individually measured kinetochores. Statistical analysis was performed with a nonparametric t test comparing two unpaired groups (Mann–Whitney test). Symbols indicate: n.s. = p > 0.05, ∗ = p ≤ 0.05, ∗∗ = p ≤ 0.01, ∗∗∗ = p ≤ 0.001, ∗∗∗∗ = p ≤ 0.0001. Red bars represent median and interquartile range.
