# Supplementary material for: RZZ‐Spindly and CENP‐E form an integrated platform to recruit dynein to the kinetochore corona
Source: EMBO J. 2023 Nov 20;42(24):e114838. doi: 10.15252/embj.2023114838 (PMC10711656; doi:10.15252/embj.2023114838)
Supplement: Supplementary file 2 — Expanded View Figures PDF [file EMBJ-42-e114838-s001.pdf]

Expanded View Figures

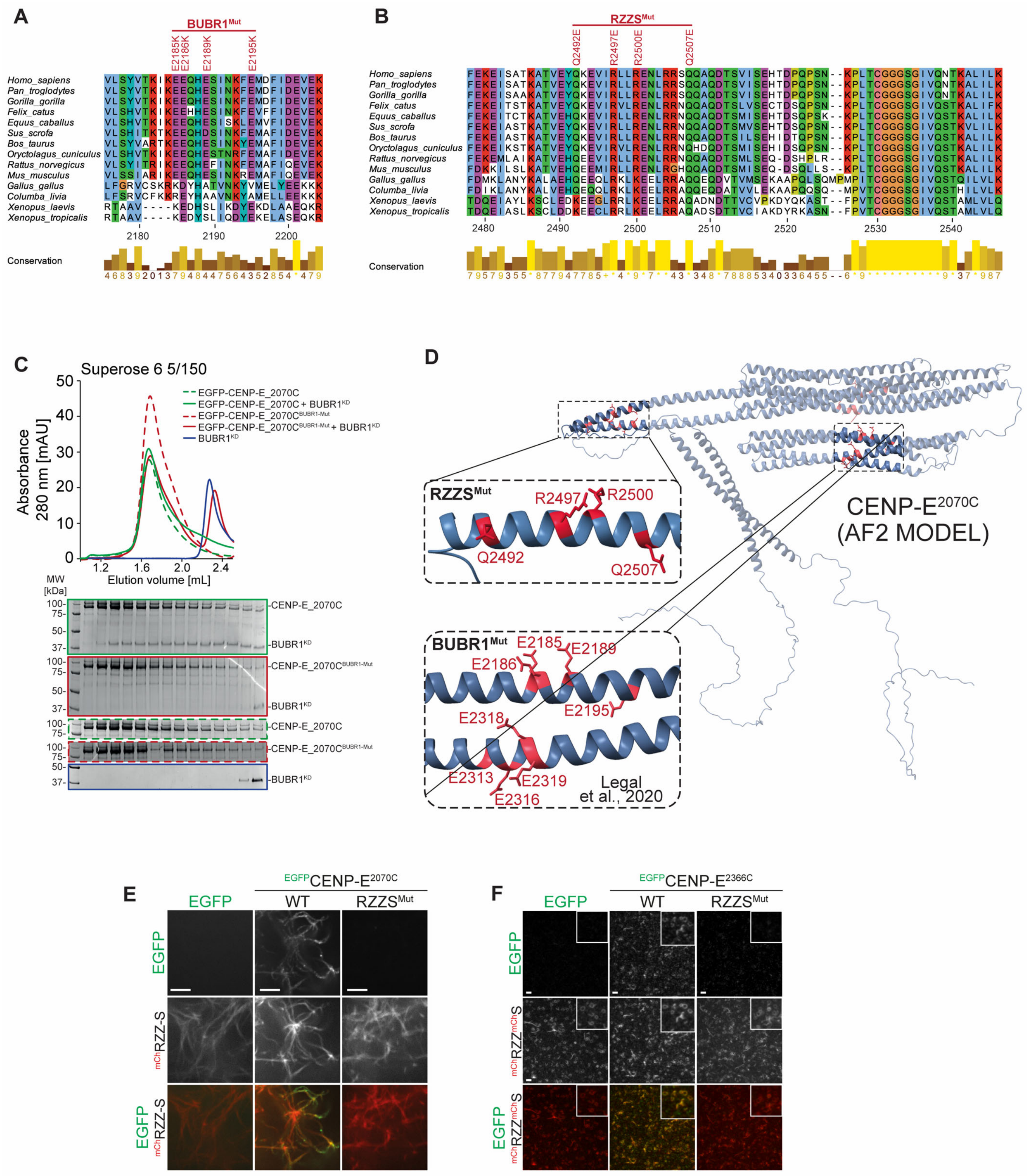

Figure EV1.

 © 2023 The Authors

Figure EV1. Identification of RZZS and BUBR1 binding sites on CENP-E.

A, B Multiple sequence alignment showing the kinetochore binding region of CENP-E was generated in Jalview with the MAFFT algorithm. Residues are depicted according to CLUSTAL color code. Amino acid substitutions comprised in the BUBR1^{Mut} and RZZS^{Mut} are labeled in red above the sequence alignment.

C Analytical SEC binding assays between the BUBR1 kinase domain (KD) and different ^{EGFP}CENP-E^{2070C} constructs. The complex run is represented as a continuous line, and the individual CENP-E constructs with a dashed line. BUBR1: 8 µM, CENP-E constructs: 16 µM. The experiment was performed once.

D AF2 Multimer model of CENP-E^{2070C}. Insets show the BUBR1^{Mut} and RZZS^{Mut} (a previously published BUBR1^{Mut}) (Legal *et al*, 2020) and surrounding sequence. The main chain is depicted in blue, and mutated residues in red.

E RZZS filament-binding assay showing recruitment of ^{EGFP}CENP-E^{2070C} constructs to ^{mCh}RZZS filaments. Scale bar: 5 µm. The experiment was performed once.

F RZZS ring-binding assay showing recruitment of ^{EGFP}CENP-E^{2070C} constructs to ^{mCH}RZZ^{mCh}S rings. The experiment was performed once. Scale bar: 5 µm.

Figure EV2. Mutual dependencies of RZZS and CENP-E and MAD1 localization.

A Schematic of the cell synchronization and imaging experiment shown in panel B.

B Representative images showing the localization of Zwilch in prometaphase after depletion of CENP-E with 60 nM siRNA. Eight hours after RNAi treatment, HeLa cells were synchronized in the G2 phase with 9 µM RO3306 for 15 h and then released into mitosis. Subsequently, cells were immediately treated with 3.3 µM Nocodazole, 10 µM MG132 and, where indicated, with 500 nM reversine, for an additional hour. CENP-C was used to visualize kinetochores and DAPI to stain DNA. Three biological replicates were performed. Scale bar: 5 µm.

C, D Quantification of Zwilch and CENP-E levels at kinetochores of the experiment shown in panel B. *n* refers to individually measured kinetochores.

E Representative images showing the localization of Zwilch in prometaphase after depletion of CENP-E with 60 nM siRNA. Thirteen hours after RNAi treatment HeLa cells were electroporated with electroporation buffer or ^{EGFP}CENP-E^{2366C}. Following an 8 h recovery, cells were synchronized in the G2 phase with 9 µM RO3306 for 15 h and then released into mitosis. Subsequently, cells were immediately treated with 3.3 µM Nocodazole, 10 µM MG132 and, where indicated, with 500 nM reversine, for an additional hour. CENP-C was used to visualize kinetochores and DAPI to stain DNA. Three biological replicates were performed. Scale bar: 5 µm. The DMSO control in the upper raw is duplicated in Appendix Fig S2F.

F, G Quantification of EGFP and Zwilch levels at kinetochores of the experiment shown in panel E. *n* refers to individually measured kinetochores.

H Representative images showing the localization of MAD1 in RPE-1 CENP-E^{AID_3xFLAG} cells treated as shown in Fig 3B. This mount is part of a larger experiment in which Zwilch was also visualized (in Fig 3D; omitted here). Therefore, the images in the CENP-C and DAPI channels are duplicates of those shown in Fig 3D, where MAD1 was instead omitted.

I Quantification of MAD1 levels at kinetochores of the experiment shown in panel H. *n* refers to individually measured kinetochores.

J Representative images showing the localization of MAD1 after inhibition of MPS1. Thirty-two hours after seeding, HeLa cells were synchronized in the G2 phase with 9 µM RO3306 for 15 h and then released into mitosis. Subsequently, cells were immediately treated with 3.3 µM Nocodazole, 10 µM MG132 and, where indicated, with 500 nM reversine, for an additional hour. CENP-C was used to visualize kinetochores and DAPI to stain DNA. Three biological replicates were performed. Scale bar: 5 µm.

K, L Quantification of CENP-E and MAD1 levels at kinetochores of the experiment shown in panel J. *n* refers to individually measured kinetochores. Red bars represent the median and interquartile range.

Data information: Statistical analysis (D, E, F, G, and I) was performed with a nonparametric *t*-test comparing two unpaired groups (Mann–Whitney test). Symbols indicate: $^{**}P \le 0.01$, $^{***}P \le 0.001$, $^{****}P \le 0.0001$. Red bars represent the median and interquartile range.

© 2023 The Authors

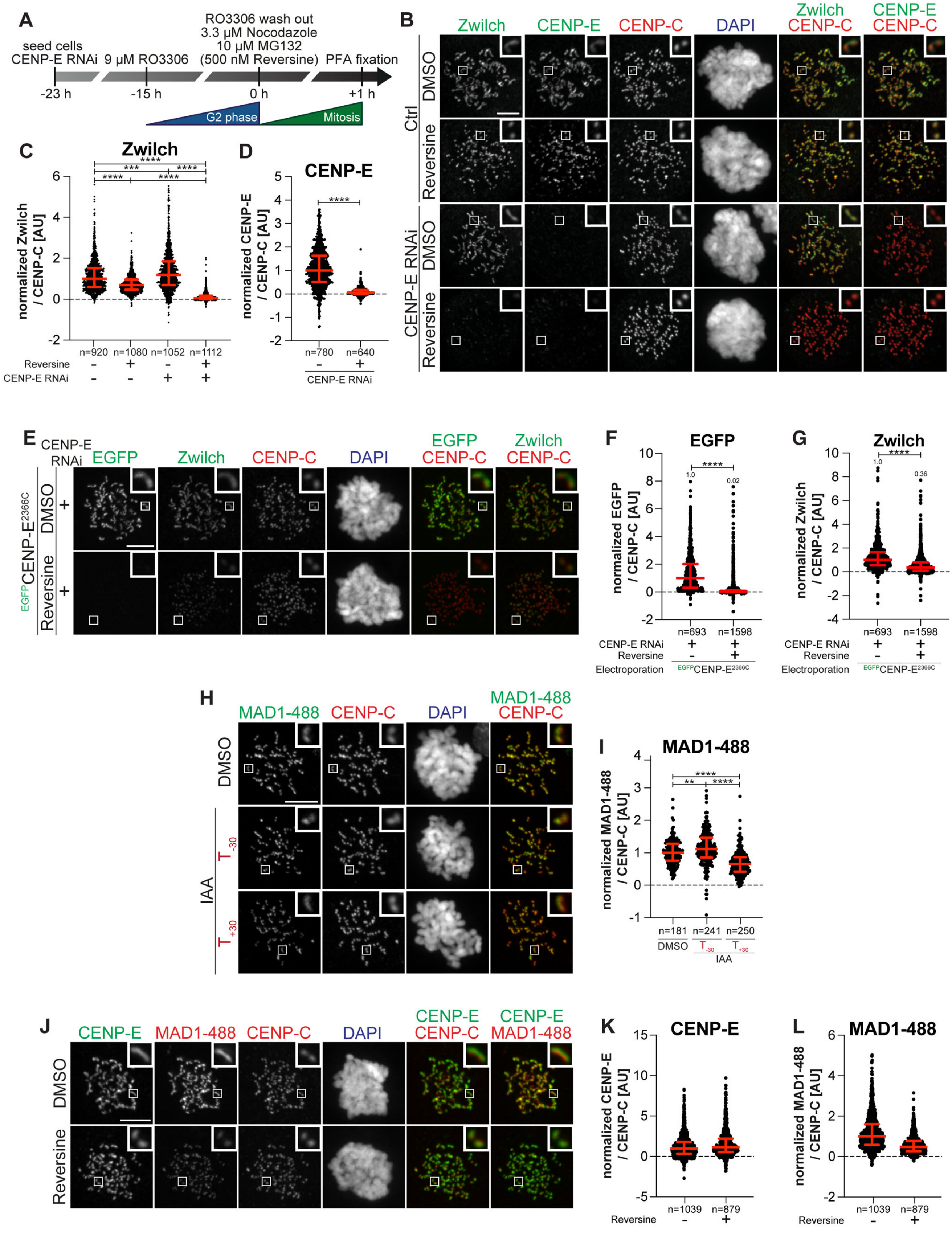

Figure EV2.

© 2023 The Authors

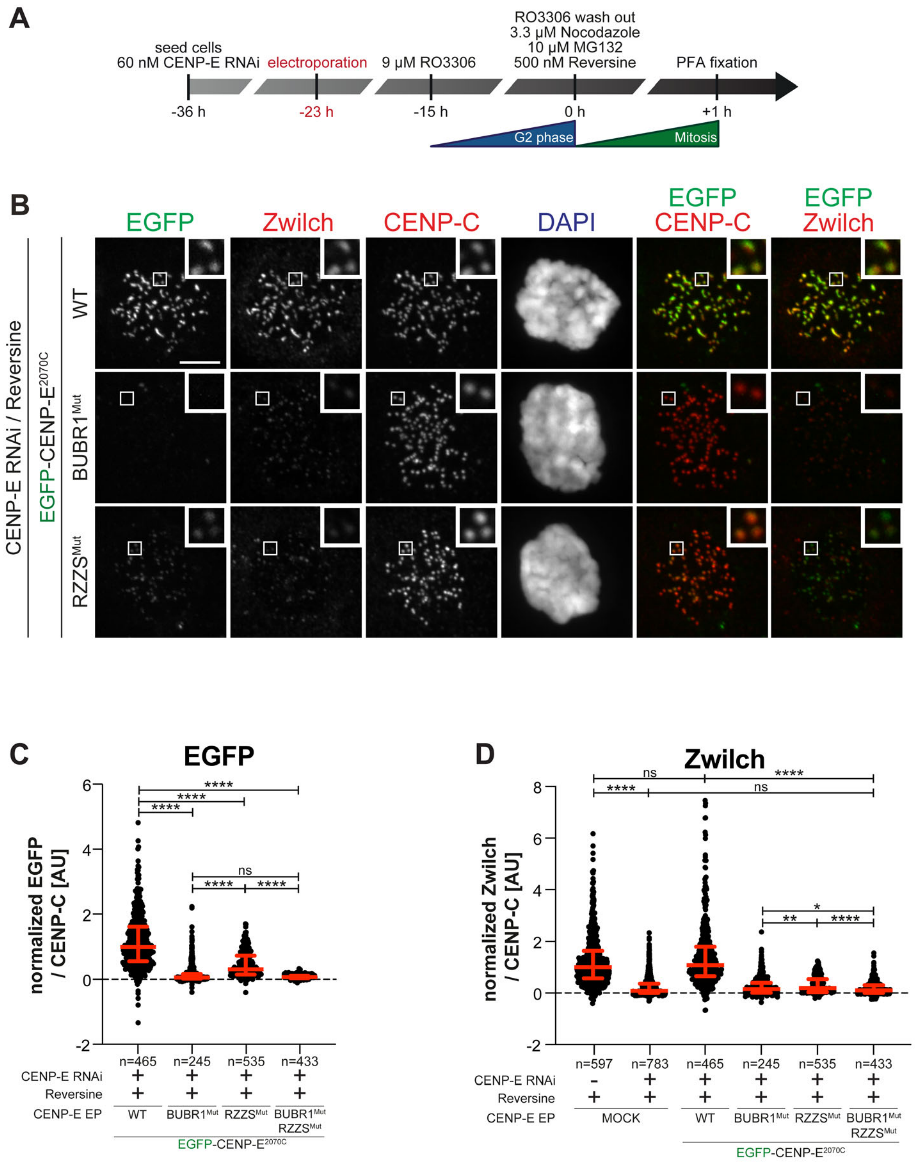

Figure EV3. CENP-E mutants require MPS1 for robust localization.

A Schematic representation of the cell synchronization protocols for the experiment in panel B.

B Representative images showing the localization of different ^{EGFP}CENP-E^{2070C} constructs in prometaphase after depletion of CENP-E with 60 nM siRNA. Thirteen hours after RNAi treatment cells were electroporated with recombinant ^{EGFP}CENP-E^{2070C} constructs as indicated. Following an 8 h recovery, cells were synchronized in the G2 phase with 9 μM RO3306 for 15 h and then released into mitosis. Subsequently, cells were immediately treated with 3.3 μM Nocodazole, 10 μM MG132 and, where indicated, with 500 nM reversine, for an additional hour. CENP-C was used to visualize kinetochores and DAPI to stain DNA. The experiment was performed once. Scale bar: 5 μm.

C, D Quantification of EGFP and Zwilch levels at kinetochores of the experiment shown in panel B. *n* refers to individually measured kinetochores.

Data information: Statistical analysis was performed with a nonparametric *t*-test comparing two unpaired groups (Mann–Whitney test). Symbols indicate: $^{n.s.}P > 0.05$, $*P \leq 0.05$, $**P \leq 0.01$, $****P \leq 0.0001$. Red bars represent the median and interquartile range.

Figure EV4. Kinetochore recruitment of RZZS requires MPS1 kinase activity.

A Representative images showing HeLa cells electroporated with the indicated ^{mCh}RZZ construct. Before fixation, cells were synchronized in the G2 phase with 9 μM RO3306 for 15 h and then released into mitosis. Subsequently, cells were immediately treated with 10 μM MG132 for an additional hour. CENP-C was used to visualize kinetochores and DAPI to stain DNA. Three biological replicates were performed. Scale bar: 5 μm.

B Quantification of Zwilch levels at kinetochores of the experiment shown in panel A. *n* refers to individually measured kinetochores.

C Representative images showing HeLa cells treated as in panel A.

D Quantification of Zwilch levels at kinetochores of the experiment shown in panel C. *n* refers to individually measured kinetochores.

E Representative images showing the localization of the indicated ^{mCh}RZZ constructs in prometaphase after depletion of Zwilch with 100 nM. Sixty-one hours after Zwilch RNAi treatment HeLa cells were electroporated with mCherry or different ^{mCh}RZZ constructs as indicated. Following an 8 h recovery, cells were synchronized in the G2 phase with 9 μM RO3306 for 15 h and then released into mitosis. Subsequently, cells were immediately treated with 3.3 μM Nocodazole for an additional hour. CENP-C was used to visualize kinetochores and DAPI to stain DNA. Three biological replicates were performed. Scale bar: 5 μm.

F Quantification of Zwilch levels at kinetochores of the experiment shown in panel E. *n* refers to individually measured kinetochores.

G Representative images showing the localization of the indicated ^{mCh}RZZ constructs in prometaphase after depletion of CENP-E with 60 nM siRNA and Zwilch with 100 nM, as shown in (Fig 4C). Thirteen hours after CENP-E RNAi treatment HeLa cells were electroporated with different ^{mCh}RZZ constructs as indicated. Following an 8 h recovery, cells were synchronized in the G2 phase with 9 μM RO3306 for 15 h and then released into mitosis. Subsequently, cells were immediately treated with 3.3 μM Nocodazole, 10 μM MG132 and, where indicated, with 500 nM reversine, for an additional hour. CENP-C was used to visualize kinetochores and DAPI to stain DNA. Two biological replicates were performed. Scale bar: 5 μm.

H, I Quantification of Zwilch levels at kinetochores of the experiment shown in panel G. *n* refers to individually measured kinetochores.

Data information: Statistical analysis (B, D) was performed with a nonparametric *t*-test comparing two unpaired groups (Mann–Whitney test). Symbols indicate: $^{n.s.}P > 0.05$, $^{*}P \leq 0.05$, $^{**}P \leq 0.01$, $^{***}P \leq 0.001$, $^{****}P \leq 0.0001$. Red bars represent the median and interquartile range. Statistical analysis (F, H, I) was performed with a nonparametric *t*-test comparing two unpaired groups (Mann–Whitney test). Symbols indicate: $^{n.s.}P > 0.05$, $^{**}P \leq 0.01$, $^{***}P \leq 0.001$, $^{****}P \leq 0.0001$. Red bars represent the median and interquartile range.

© 2023 The Authors

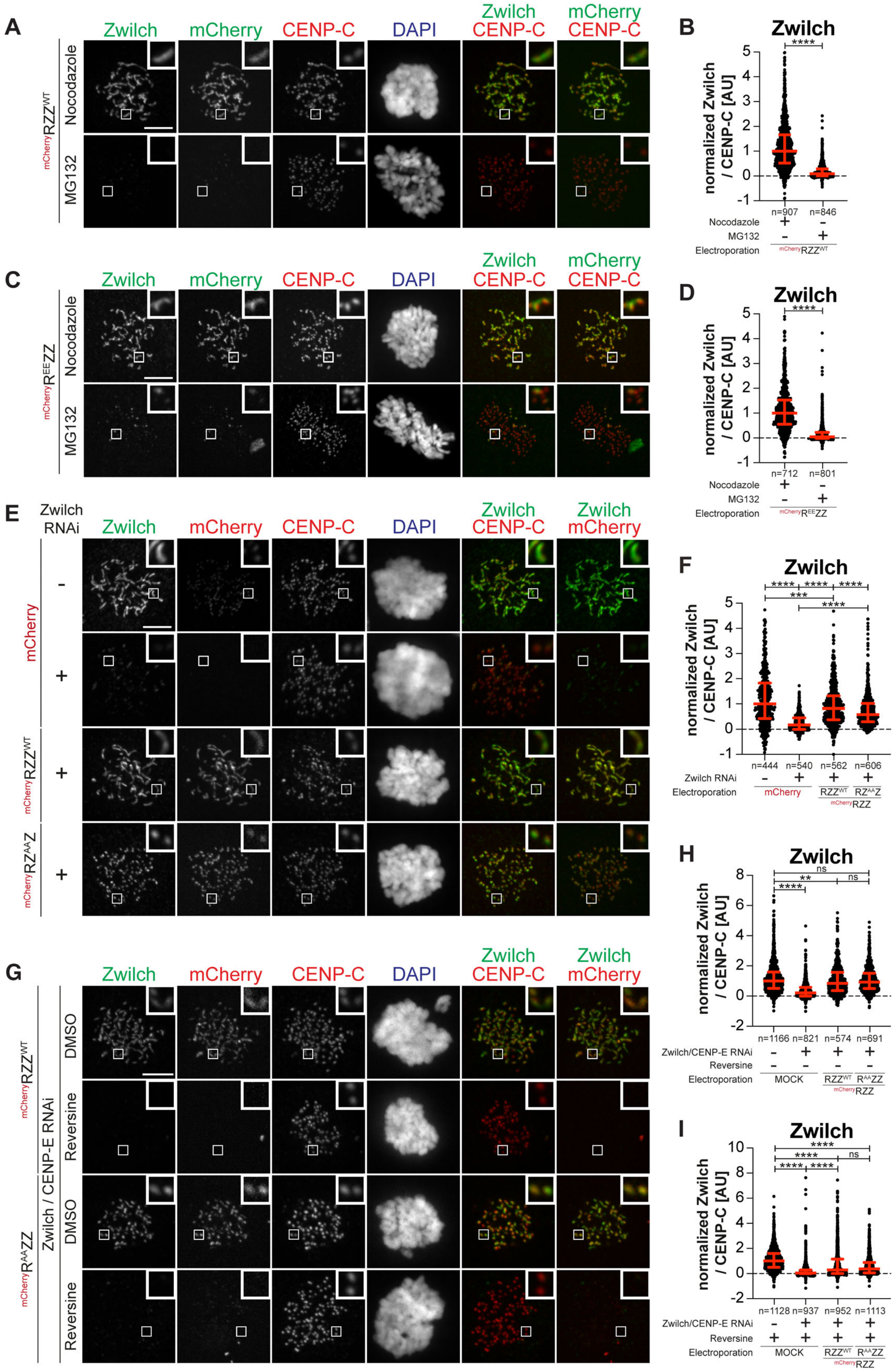

A
Zwilch
mCherry
CENP-C
DAPI
Zwilch CENP-C
mCherry CENP-C
mCherryRZZWT
Nocodazole
MG132
B
Zwilch
normalized Zwilch / CENP-C [AU]
****
n=907 n=846
Nocodazole
MG132
Electroporation
C
mCherryREEZZ
D
Zwilch
n=712 n=801
E
Zwilch RNAi
mCherry
mCherryRZZWT
mCherryRZAAZ
F
Zwilch
n=444 n=540 n=562 n=606
Zwilch RNAi
Electroporation
G
mCherryRAAZZ
Zwilch / CENP-E RNAi
DMSO
Reversine
H
Zwilch
n=1166 n=821 n=574 n=691
Zwilch/CENP-E RNAi
Reversine
Electroporation
MOCK
I
Zwilch
n=1128 n=937 n=952 n=1113

Figure EV4.

Figure EV5. Characterization of Spindly autoinhibition.

A AF2 Multimer model of Spindly$^{1-309}$ (d'Amico *et al*, 2022) and multiple sequence alignment showing the CC2 region of Spindly was generated in Jalview. Residues are depicted according to CLUSTAL color code. Amino acid substitutions mutated in the Spindly^{4A} construct are labeled in magenta above the sequence alignment. The inset in the AF2 model shows amino acids 275–306 of Spindly and surrounding sequence. The main chain is depicted in green and mutated residues in magenta.

B, C Analytical SEC binding assays between the dynactin-PE (brown) and ^{mCh}Spindly constructs. The complex run is represented as a continuous line, and the individual Spindly constructs with a dashed line. PE: 3 μM, Spindly constructs: 16 μM. The control gels with dynactin-PE alone are shared between panels B and C. The experiment was performed twice.

D, E Analytical SEC binding assays between the CENP-E^{2070C} and ^{mCh}Spindly constructs. The complex run is represented as continuous line and the individual constructs with a dashed line. CENP-E: 20 μM, Spindly constructs: 16 μM. The control gels with ^{mCh}Spindly^{4A} alone are shared between panels C and E. The experiment was performed twice.

F, G Quantification of dynactin-p150^{glued} and mCherry levels at kinetochores after depletion of CENP-E with 60 nM siRNA and Spindly with 50 nM siRNA. Thirteen hours after CENP-E RNAi treatment cells were electroporated with electroporation buffer or recombinant ^{mCh}Spindly constructs as indicated. Following an 8 h recovery, cells were synchronized in the G2 phase with 9 μM RO3306 for 15 h and then released into mitosis. Subsequently, cells were immediately treated with 3.3 μM Nocodazole for an additional hour. *n* refers to individually measured kinetochores.

H Representative images showing the localization of dynactin monitored through the p150^{glued} subunit after depletion of CENP-E with 60 nM siRNA and CENP-F with 50 nM. Eight hours after RNAi treatment, HeLa cells were synchronized in the G2 phase with 9 μM RO3306 for 15 h and then released into mitosis. Subsequently, cells were immediately treated with 3.3 μM Nocodazole for an additional hour. CENP-C was used to visualize kinetochores and DAPI to stain DNA. Three biological replicates were performed. Scale bar: 5 μm.

I–K Quantification of CENP-E, CENP-F and dynactin-p150^{glued} levels at kinetochores of the experiment shown in panel H. *n* refers to individually measured kinetochores.

Data information: Statistical analysis (F, G, I–K) was performed with a nonparametric *t*-test comparing two unpaired groups (Mann–Whitney test). Symbols indicate: $^{n.s.}P > 0.05$, $^{*}P \leq 0.05$, $^{***}P \leq 0.001$, $^{****}P \leq 0.0001$. Red bars represent the median and interquartile range. Three biological replicates were performed.

 © 2023 The Authors

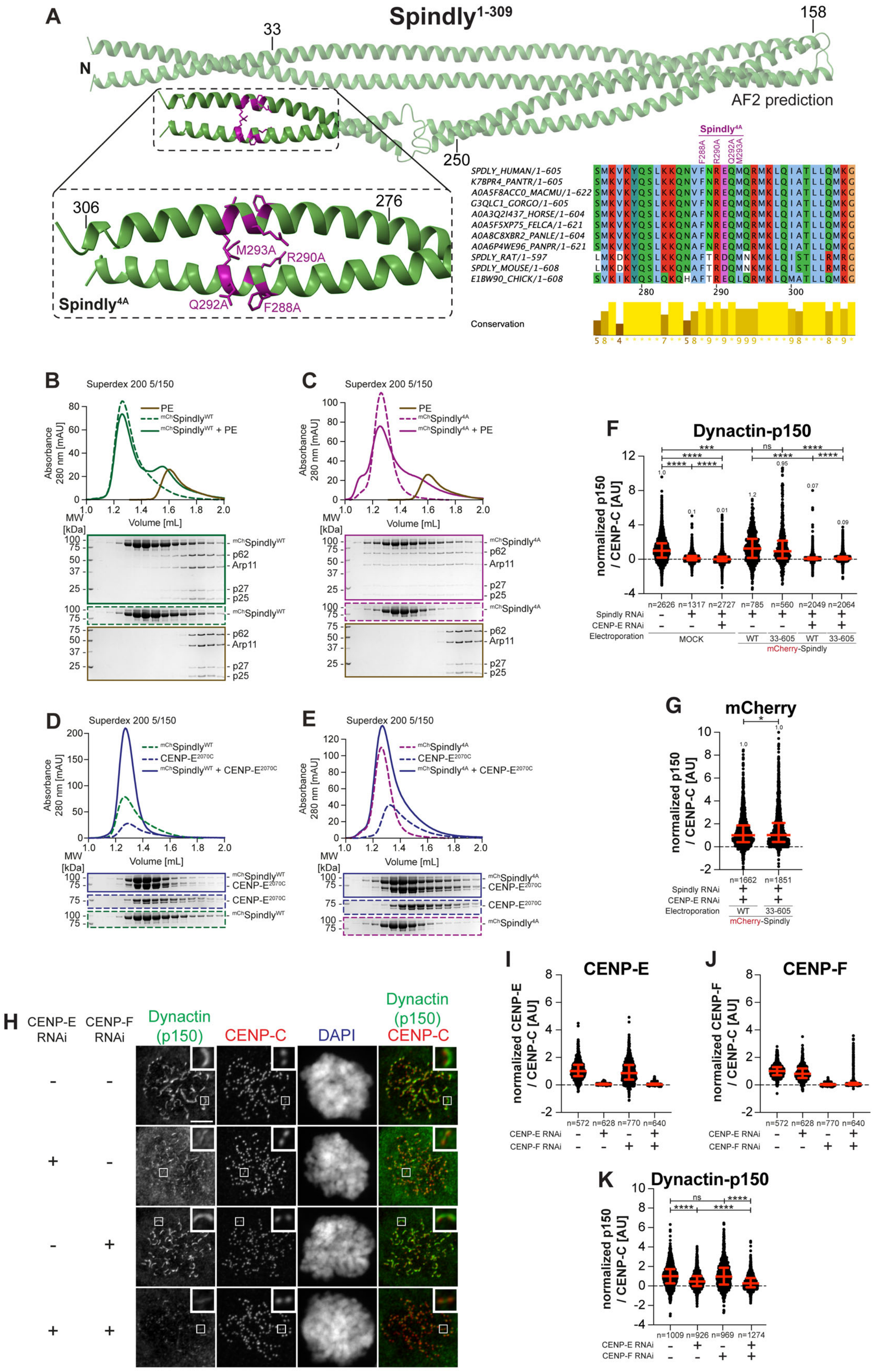
Spindly1-309
AF2 prediction
Spindly4A
Dynactin-p150
mCherry
CENP-E
CENP-F
Dynactin-p150

Figure EV5.
