# Supplementary material for: RZZ‐Spindly and CENP‐E form an integrated platform to recruit dynein to the kinetochore corona
Source: EMBO J. 2023 Nov 20;42(24):e114838. doi: 10.15252/embj.2023114838 (PMC10711656; doi:10.15252/embj.2023114838)
Supplement: Supplementary file 4 [file EMBJ-42-e114838-s002.pdf]

THE EMBO JOURNAL

RZZ-Spindly and CENP-E form an integrated platform to recruit Dynein to the kinetochore corona

Andrea Musacchio, Verena Cmentowski, Giuseppe Ciossani, Ennio d'Amico, Sabine Wohlgemuth, Brian Dynlacht, and Mikito Owa

DOI: 10.15252/embj.2023114838

Corresponding author(s): Andrea Musacchio (andrea.musacchio@mpi-dortmund.mpg.de) , Verena Cmentowski (verena.cmentowski@mpi-dortmund.mpg.de)

Review Timeline:

Submission Date:	24th Jun 23
Editorial Decision:	2nd Aug 23
Revision Received:	27th Sep 23
Editorial Decision:	24th Oct 23
Revision Received:	25th Oct 23
Accepted:	26th Oct 23

Editor: Ieva Gailite/Hartmut Vodermaier

Transaction Report:

(Note: With the exception of the correction of typographical or spelling errors that could be a source of ambiguity, letters and reports are not edited. Depending on transfer agreements, referee reports obtained elsewhere may or may not be included in this compilation. Referee reports are anonymous unless the Referee chooses to sign their reports.)

1st Editorial Decision **2nd Aug 2023**

Dear Dr. Musacchio,

Thank you for submitting your manuscript for consideration by the EMBO Journal. We have now received comments from three reviewers, which I am sharing with you on behalf of my colleague Hartmut Vodermaier, who is away from the office at the moment.

As you will see from the reports, all reviewers appreciate the study, while indicating several reasonable points that could be improved in the revised version. Based on these positive comments, I invite you to submit a revised version of the manuscript. I would suggest to contact Hartmut for further discussion of the revision upon his return to the office on August 16.

We generally allow three months as standard revision time. As a matter of policy, competing manuscripts published during this period will not negatively impact on our assessment of the conceptual advance presented by your study. However, please contact me as soon as possible upon publication of any related work to discuss the appropriate course of action. Should you foresee a problem in meeting this deadline, please let us know in advance to discuss an extension.

When preparing your letter of response to the referees' comments, please bear in mind that this will form part of the Review Process File and will therefore be available online to the community. For more details on our Transparent Editorial Process, please visit our website: https://www.embopress.org/page/journal/14602075/authorguide#transparentprocess. Please also see the attached instructions for further guidelines on preparation of the revised manuscript.

Please feel free to contact me or Hartmut if you have any further questions regarding the revision. Thank you for the opportunity to consider your work for publication, and we look forward to receiving the revised manuscript.

With best regards,

Ieva

Ieva Gailite, PhD
Senior Scientific Editor
The EMBO Journal
Meyerhofstrasse 1
D-69117 Heidelberg
Tel: +4962218891309
i.gailite@embojournal.org

Instructions for preparing your revised manuscript:

Please make sure you upload a letter of response to the referees' comments together with the revised manuscript.

Please also check that the title and abstract of the manuscript are brief, yet explicit, even to non-specialists.

When assembling figures, please refer to our figure preparation guideline in order to ensure proper formatting and readability in print as well as on screen:
https://bit.ly/EMBOPressFigurePreparationGuideline
See also guidelines for figure legends: https://www.embopress.org/page/journal/14602075/authorguide#figureformat

At EMBO Press we ask authors to provide source data for the main manuscript figures. Our source data coordinator will contact you to discuss which figure panels we would need source data for and will also provide you with helpful tips on how to upload and organize the files.

IMPORTANT: When you send the revision we will require
- a point-by-point response to the referees' comments, with a detailed description of the changes made (as a word file).
- a word file of the manuscript text.
- individual production quality figure files (one file per figure)
- a complete author checklist, which you can download from our author guidelines (https://www.embopress.org/page/journal/14602075/authorguide).
- Expanded View files (replacing Supplementary Information)
Please see out instructions to authors
https://www.embopress.org/page/journal/14602075/authorguide#expandedview

Please remember: Digital image enhancement is acceptable practice, as long as it accurately represents the original data and conforms to community standards. If a figure has been subjected to significant electronic manipulation, this must be noted in the figure legend or in the 'Materials and Methods' section. The editors reserve the right to request original versions of figures and the original images that were used to assemble the figure.

Further information is available in our Guide For Authors: https://www.embopress.org/page/journal/14602075/authorguide

We realize that it is difficult to revise to a specific deadline. In the interest of protecting the conceptual advance provided by the work, we recommend a revision within 3 months (31st Oct 2023). Please discuss the revision progress ahead of this time with the editor if you require more time to complete the revisions. Use the link below to submit your revision:

https://emboj.msubmit.net/cgi-bin/main.plex

Referee #1:

The molecular basis of corona assembly at kinetochores has been extremely challenging to dissect due to the number of components involved and the low affinity of individual protein-protein interactions within this ephemeral structure. How dynein-dynactin and the kinesin CENP-E are recruited to the corona is of particular interest, since these two opposing motors drive chromosome congression. Previous work established that dynein-dynactin is recruited by the adaptor Spindly, which in turn is recruited by the RZZ complex. Kinetochore-localized RZZ-Spindly oligomerizes into a filamentous meshwork, which requires phosphorylation of the Rod subunit by Mps1 kinase, but the identity of the components that initially recruit the RZZ complex to the kinetochore has not been established with any certainty. How CENP-E is recruited has also remained relatively mysterious: CENP-E binds the outer kinetochore component BubR1, yet disrupting this interaction is insufficient to prevent kinetochore localization of the motor. Here Cmentowski and colleagues present a rigorous and comprehensive dissection of the recruitment pathways for dynein-dynactin and CENP-E, using previously validated as well as newly generated separation-of-function mutants of the RZZ complex, Spindly, and CENP-E.
The authors first establish that two distinct regions in the CENP-E C-terminus recruit the motor to the outer kinetochore and the corona by interacting with BUBR1 and RZZ-Spindly, respectively. Quantitative assessment of CENP-E recruitment to kinetochores in cultured cells is complemented by a powerful in vitro reconstitution assay that reports on the ability of CENP-E to be recruited to RZZS filaments. Importantly, the authors succeed in generating CENP-E point mutants that specifically perturb the BUBR1 or RZZ-Spindly interaction. The BUBR1 recruitment pathway is shown to maintain CENP-E at the kinetochore during metaphase after the corona has been disassembled, and simultaneous perturbation of the BUBR1 and RZZ-Spindly interaction abrogates CENP-E recruitment to kinetochores altogether. Conversely, CENP-E becomes essential for RZZ-Spindly recruitment to kinetochores when RZZ-Spindly oligomerization is suppressed by Mps1 inhibition, and CENP-E additionally has a more direct role in dynein-dynactin recruitment. The co-dependence of dynein-dynactin and CENP-E for corona recruitment has significant implications for the interpretation of previous studies that used CENP-E depletion to define the role of the kinesin in chromosome congression. By electroporating cells with reconstituted RZZ complex mutants that either cannot oligomerize or are able to oligomerize independently of Mps1, the authors then uncover a role for Mps1 in RZZ recruitment and identify Knl1 as the other main kinetochore receptor for RZZ besides CENP-E.

The manuscript is written in a concise manner and the experiments are executed and presented to a high standard. The results significantly advance our understanding of corona assembly and establish the corona as a specialized structure for the pairing of opposite-polarity motors, which has implications for organelle and vesicle transport where dynein and kinesins also co-exist on the same cargo.

Specific comments:

- Figure 6 & Figure 6 - Supplement 1: an important conclusion of the study is that Knl1 is the core kinetochore receptor of RZZ. Indeed, the authors show that Knl1 depletion on its own already significantly decreases kinetochore levels of RZZ in HeLa cells, which agrees with prior studies in this cell line. However, prior studies in RPE1 cells reported little or no effect of Knl1 depletion on RZZ recruitment to kinetochores (Silió et al., 2015; Rodriguez-Rodriguez et al., 2018), which challenges the notion that Knl1 is the core receptor for RZZ in this diploid cell line. The authors have the perfect tools to clarify this important point using a similar strategy as for HeLa cells, i.e deplete Knl1 by RNAi (as done by Silió et al., 2015) in the RPE-1 CENP-E AID background and electroporate in the R(EE)ZZ mutant. If Knl1 also acts as the core receptor for RZZ in RPE1 cells, RZZ should disappear from kinetochores in this condition.

- Figure 2 - Supplement 1: Readers may not readily understand the difference between RZZS filaments (E) and mini-circles (F). As far as this reviewer understands, RZZS filaments are generated by mCherry-ROD/Zw10/Zwlich/Spindly, whereas mini-circles form when Spindly is additionally tagged with mCherry. If this is so, the label for RZZS in (E) needs to be changed. Also, why is

EGFP alone recruited to mini-circles in (F)?

- Since different cell lines are used throughout the study, it would be helpful to indicate the specific cell line used for each experiment in the figure legends (this currently not the case for all legends).

Referee #2:

The manuscript from Cmentowski et al. reports a number of interesting and important observations on motor proteins in the fibrous corona of the kinetochore in human cells. This effort continues a long-standing effort from the Musacchio group to reconstitute and understand the molecular interactions underlying formation and function of distinct sub-domains of the human kinetochore. Overall, the work is executed at a technically impressive level and the manuscript written with scholarly depth to place the findings in the context of the literature. While I am very supportive of the study, I did find it difficult to navigate the many different threads and a lack of differentiation between rigorously established findings and more speculative views on questions that will require future work to elucidate. The work also does not directly investigate the functional significance of the newly defined interactions beyond kinetochore localization analysis in nocodazole (note that the authors ability to rescue by electroporation with high quality recombinant proteins/complexes makes the localization analysis exceptionally rigorous - this is a strong point of the paper). Given the scope and rigor of work presented, the authors do not need to conduct any further functional analysis but to motivate others to take on this challenge it is important that the major findings be presented in a manner that is accessible and impactful in guiding the field.

Major comments:

The manuscript presents 3 clear findings and the comments below are focused on each finding along with requests for clarification and suggestions on presentation:

i) The C-terminus of CENP-E contains independent sites for interaction with BubR1 (known from previous work) and RZZS; the BubR1 interaction is important for retention of CENP-E at aligned chromosomes and the RZZS interaction is important for CENP-E corona localization. Disrupting the RZZS interaction or both interactions negates the dominant-negative effect of over-expressing a C-terminal CENP-E fragment on chromosome alignment. These data shown in Figs 1 & 2 and associated supplemental figures are outstanding. With respect to the RZZS interaction, which is the major new finding here, have the authors tested direct interaction with the C-terminal CENP-E fragment using their gel filtration approach? Fig. 1H & I and the supplement presents TIRF-based analysis of interaction of the CENP-E C-terminal fragment with RZZS filaments and RZZS minicircles but not gel filtration (likely has been attempted and, even if negative, should be commented on). The authors should emphasize the new interaction they identify (CENP-E with RZZS) by potentially moving their mutant design disrupting this interaction to the primary figures (the structural model in Fig. S1D is very compelling and should be a primary figure panel). Their one piece of functional analysis also strongly suggests that the RZZS-bound pool of CENP-E in the corona is important for chromosome alignment (comparing Fig. 2A to Fig. 2I, RZZS-Mut is only slightly better than BubR1-Mut in the localization analysis but significantly worse than BubR1-mut in the chromosome alignment analysis; this point is not coming through in part because these two figure panels are split apart - they should be presented adjacent to each other). In addition, the FL CENP-E appears less sensitive to individual interaction perturbations than the C-terminal fragment (comparing Fig. 2A,B to Fig. 2G,H) - this should be commented on.

ii) Mps1 inhibition affects corona assembly (shown previously) but only modestly affects RZZS kinetochore localization; however, combining Mps1 inhibition with CENP-E depletion (which on its own has no significant effect on RZZS localization) leads to loss of RZZS from kinetochores. This is an important finding that is completely unexpected. The data are strong and conclusions are well supported. One prediction, that would help link these results to the first part of the manuscript, would be to analyze BubR1mut and RZZSmut CENP-E with Mps1 inhibition. Would both synergize with Mps1 inhibition in affecting RZZS localization? The authors do present data on the 2366C fragment of CENP-E (which lacks the BubR1 interaction site) but directly testing the mutants defined in the first part would help connect these two parts of the manuscript together. The figure would also be easier to interpret if the single Mps1 inhibition, shown in the supplement, was featured in the primary figure.

The authors next focus on dynein-dynactin localization (point iii below) but then follow with a section on how mimicking Mps1 phosphorylation of RZZ (specifically of ROD) bypasses the CENP-E & Mps1 activity requirement. It would be best if this section was moved up and immediately followed the rest of point ii - they are logically connected. One conclusion from this section is that corona assembly does not require CENP-E or Mps1 activity if the two sites in Rod are phosphomimetic, highlighting the existence of a corona "receptor" that remains to be defined.

iii) CENP-E is important for dynein-dynactin recruitment to the corona. Despite over 3 decades of work on these two motors localized to kinetochores, it is remarkable that this relationship between them was missed (at least I was unable to find a simple deplete & localize experiment similar to what is reported here in prior literature). Note that Thrower et al. in the mid-90s had purified a minus end-directed motor activity associated with CENP-E but concluded that this activity was not dynein (PMID: 7889940); in hindsight, it seems likely that the activity may in fact be dynein (motility rate observed was consistent with that), which could be revisited with current mass spec and other approaches. The authors suggest that mutating the RZZS interface of

CENP-E compromises its ability to recruit dynein-dynactin - however, as the RZZS mutant itself localizes worse than the WT 2070C fragment of CENP-E (Fig. 2A), this conclusion should be re-assessed after normalizing for the difference between WT and RZZS-Mut localization; they also show, based on prior work, that residual dynein observed is likely being recruited by CENP-F. The authors test Spindly mutants that relieve its auto-inhibition and conclude that CENP-E does not act by relieving Spindly auto-inhibition (acknowledging that the mutants may not be "fully open"). Thus, the precise molecular reason for why CENP-E is needed to recruit dynein-dynactin remains unclear. Once again, comparing WT, BubR1-mut and RZZS-mut CENP-E 2070C fragments for dynein-dynactin recruitment (normalizing for their own localization) may help tie the manuscript together - this is a suggestion rather than a requirement for revision and left to the authors' discretion.

My strong recommendation to the authors is to end the manuscript at this point. The subsequent analysis, which does not lead to similarly strong conclusions, only serves to add complexity, dilute the impact of the findings above, and has caveats (all of which are properly acknowledged). These latter points are best saved for future manuscripts and leaving them out will in fact enhance the impact of the advances made by the authors. By taking some data/schematics from the supplement and integrating them into the manuscript structure, the authors will have presented an important and accessible contribution to addressing the relationship between the two key motor proteins at the kinetochore. A related suggestion would be for them to present a model figure that highlights the 3 key advances above and leave more complex schematics aimed at integration for a future review.

Referee #3:

In this paper, Cmentowski and colleagues present a characterization of a molecular interaction network involving multiple components of the kinetochore corona, detailing both its assembly and its expansion mechanisms. Corona is a transient structure that assembles on the outer kinetochore during early mitosis and is essential for timely and error-free chromosome bi-orientation, alignment, and microtubule nucleation from kinetochores. Corona also serves as a platform for timely activation and deactivation of the spindle assembly checkpoint. Although highly studied in both functional and structural sense during the last decade, the understanding of detailed interaction networks that are responsible for corona assembly, maintenance, and expansion is still hampered by the large number of components involved and their complex molecular and regulatory interconnectivity, including, among others, the RZZ complex and Spindly (RZZS), CENP-E, CENP-F, Dynein-Dynactin (DD), Mad1:Mad2 complex and numerous kinases and phosphatases of which MPS1 kinase seems to play a central role in corona assembly. To battle with this complexity of interactions within the corona, the authors used a large set of state-of-the-art separation mutants of multiple corona components, most of which were developed in this paper, together with classic depletion approaches by RNA interference and auxin-inducible protein degradation. First, the authors show that CENP-E, a motor protein that resides in the kinetochore corona and mediates chromosome alignment, interacts not only with BUBR1, as previously shown, but also with the RZZ complex, and does so by using distinct regions. The authors were able to determine exact residues responsible for the interaction of CENP-E with both the BUBR1 and RZZ complex and further mutate these regions to establish a specific separation of CENP-E function mutants. They showed that mutants are localized to different parts of the kinetochore substructure, the outer kinetochore for BUBR1, and the corona for the RZZ-binding region. Thus, the authors showed that these distinct regions independently promote the recruitment of CENP-E to the kinetochore. The authors also show that after the establishment of biorientation and the loss of the corona, BUBR1 is the only receptor that mediates CENP-E localization on the kinetochore. Furthermore, the authors show that CENP-E is involved in the recruitment of RZZS to the kinetochore. However, CENP-E is not required for the recruitment of RZZs to the corona when the structure is preestablished, but is instead essential for the recruitment of RZZs when the corona assembly is perturbed by inhibition of MPS1. Furthermore, the authors showed that CENP-E contributes to the accumulation of Dynein-Dynactin to the kinetochore, presumably independently of the well-characterized DD kinetochore adaptor Spindly, together with the corona protein CENP-F. The authors further confirm that the corona can assemble without CENP-E by exploiting RZZ phosphomimetic mutants that mimic MPS1 phosphorylation of the ROD protein. By using additional separation of function mutants of the RZZ complex and Spindly depletion, both of which block corona expansion independently of MPS1, the authors were able to show that MPS1 is involved in the recruitment of RZZs to the kinetochore independently of its function in corona expansion, suggesting an additional kinetochore receptor for RZZs in addition to CENP-E. Finally, the authors were able to show through the use of RZZ phosphomimetic mutants that the kinetochore protein KNL1, either directly or indirectly, plays an important role in the recruitment of the RZZ complex, with a minor role of NDC80 and BUB1.
I think this is a highly valuable study that explores and substantially advances our knowledge on one of the most fundamental aspects of kinetochore function during early mitosis, namely corona assembly and shedding. I believe that the study will be highly relevant for researchers studying signaling during cell division, mechanisms of chromosome alignment, molecular motors, and the structure of the kinetochore. What I would like to emphasize as a strong feature of this paper is its thorough use of various separation-of-function mutants to pinpoint the molecular determinants of the localization of various interdependent components within this highly complex structure. In that regard, it is my opinion that the approach presented here could be used as a role model of how similarly complex interaction networks within cells could be studied. The manuscript is written in a clear style, the figures are presented in such a detailed way, the experimental logic behind each hypothesis is elaborated, and the control experiments were carried out in such a rigorous way that I can only congratulate the authors for their work on this 'dauntingly complex process.' Therefore, it is my opinion that the manuscript is acceptable for publication in the EMBO Journal without the need for a major revision. I have, however, few minor comments that I depict below, in which I suggest the authors to elaborate more on certain points, or reanalyze some of the data to make some points within the text clearer.

Minor points:
1) The title of the manuscript in its current form is a little bit vague, and I would suggest trying something more specific as the title. This is due to the use of the "integration of microtubule motors of opposite polarity", which seems a bit premature to state here, in my opinion, since this scaffold would presumably influence the function of these proteins in chromosome biorientation, alignment, and SAC satisfaction, and for testing that the manuscript is currently lacking more direct functional approaches.
2) It would be useful for the authors to quantify the level of colocalization of the CENP-E mutants BUBR1Mut and RZZSMut with Zwilch and CENP-C in the experiment presented in Figure 2A-C. This would substantiate the notion that the BUBR1 region is mainly mediated by the kinetochore localization of CENP-E, presumably implying a strong colocalization with CENP-C and less strong with Zwilch, and exactly the opposite for the RZZS region decorating more the corona region. See also point 7.
3) It is puzzling to me that the normalized signal intensities of various kinetochore components across multiple different experiments in control conditions seem to be very dispersed and in certain rare cases close to or even below the zero value, matching the low or absent signal when components were depleted or removed by other means. Is this due to low values in certain kinetochores or do whole cells sometimes have low values? I would comment on possible reasons in the figure caption or in Methods, as this seems to be a general phenomenon across different measurements.
4) The experiment that worried me the most is the one presented in Figure 2I. I did not fully understand how the authors came to the conclusion that simultaneous interaction with RZZS and BUBR1 stabilizes CENP-E from the data presented on Figure2I. The authors should first statistically compare the effects of various mutants on chromosome alignment presented now in Figure 2I. Second, I would recommend at least speculating on what the possible basis would be for the differential ability of mutants to displace endogenous CENP-E, especially since CENP-E2070C and its BUBR1Mut look very similar in terms of their effects on chromosome alignment, contrary to other two mutants. Furthermore, the authors should speculate which of these mutants would be able to restore chromosome alignment in the case endogenous CENP-E is depleted and specific FL mutants of CENP-E (BUBR1Mut and RZZSMut) are expressed with functional motor domain.
5) The authors should define the abbreviation 'CENP-EAID' when using it for the first time on page 9, similarly as the authors have done for other abbreviations in the manuscript. Currently it is first mentioned in the last paragraph on page 9, although the method was explained already in the preceding paragraph.
6) I was a little puzzled why the authors studied in such detail the possible role of CENP-E in relieving Spindly autoinhibition and DD recruitment (Figures 4E-M), when at the end of the previous paragraph the authors presented an experiment that implied that "CENP-E does not contribute to DD recruitment by controlling Spindly levels". Although I understand the logic behind this, for the ease of reading I would suggest better linking of two parts, thus explaining the logic behind auto-inhibition path more clearly.
7) On page 16, in the Discussion part of the manuscript, the authors state that "The interaction with BUBR1 is dispensable for kinetochore recruitment of CENP-E in prometaphase, but CENP-E is clearly identified in corona, but becomes essential for kinetochore recruitment of CENP-E after corona shedding...". I don't understand the logic behind this conclusion. I mean if experimentally BUBR1 is depleted, will CENP-E be present at the outer kinetochore, at the place where it colocalizes with CENP-C but not with Zwilch and corona? In other words, how much CENP-E colocalizes with CENP-C in the absence of BUBR1 or in RZZSMut, where the interaction is presumably mediated only by BUBR1? This is related to point 2, and to the theme of two pools of CENP-E the authors discussed in Discussion part of the Manuscript.
8) On page 18 when stating "While until now corona has been primarily viewed as a platform for the coordination of Dynein motility and spindle assembly checkpoint activity," I would suggest citing a review paper.

Referee #1:

The molecular basis of corona assembly at kinetochores has been extremely challenging to dissect due to the number of components involved and the low affinity of individual protein-protein interactions within this ephemeral structure. How dynein-dynactin and the kinesin CENP-E are recruited to the corona is of particular interest, since these two opposing motors drive chromosome congression. Previous work established that dynein-dynactin is recruited by the adaptor Spindly, which in turn is recruited by the RZZ complex. Kinetochore-localized RZZ-Spindly oligomerizes into a filamentous meshwork, which requires phosphorylation of the Rod subunit by Mps1 kinase, but the identity of the components that initially recruit the RZZ complex to the kinetochore has not been established with any certainty. How CENP-E is recruited has also remained relatively mysterious: CENP-E binds the outer kinetochore component BubR1, yet disrupting this interaction is insufficient to prevent kinetochore localization of the motor. Here Cmentowski and colleagues present a rigorous and comprehensive dissection of the recruitment pathways for dynein-dynactin and CENP-E, using previously validated as well as newly generated separation-of-function mutants of the RZZ complex, Spindly, and CENP-E.

The authors first establish that two distinct regions in the CENP-E C-terminus recruit the motor to the outer kinetochore and the corona by interacting with BUBR1 and RZZ-Spindly, respectively. Quantitative assessment of CENP-E recruitment to kinetochores in cultured cells is complemented by a powerful in vitro reconstitution assay that reports on the ability of CENP-E to be recruited to RZZS filaments. Importantly, the authors succeed in generating CENP-E point mutants that specifically perturb the BUBR1 or RZZ-Spindly interaction. The BUBR1 recruitment pathway is shown to maintain CENP-E at the kinetochore during metaphase after the corona has been disassembled, and simultaneous perturbation of the BUBR1 and RZZ-Spindly interaction abrogates CENP-E recruitment to kinetochores altogether. Conversely, CENP-E becomes essential for RZZ-Spindly recruitment to kinetochores when RZZ-Spindly oligomerization is suppressed by Mps1 inhibition, and CENP-E additionally has a more direct role in dynein-dynactin recruitment. The co-dependence of dynein-dynactin and CENP-E for corona recruitment has significant implications for the interpretation of previous studies that used CENP-E depletion to define the role of the kinesin in chromosome congression. By electroporating cells with reconstituted RZZ complex mutants that either cannot oligomerize or are able to oligomerize independently of Mps1, the authors then uncover a role for Mps1 in RZZ recruitment and identify Knl1 as the other main kinetochore receptor for RZZ besides CENP-E.

The manuscript is written in a concise manner and the experiments are executed and presented to a high standard. The results significantly advance our understanding of corona assembly and establish the corona as a specialized structure for the pairing of opposite-polarity motors, which has implications for organelle and vesicle transport where dynein and kinesins also co-exist on the same cargo.

We are grateful to the reviewer for this very positive assessment of our work

Specific comments:

- Figure 6 & Figure 6 - Supplement 1: an important conclusion of the study is that Knl1 is the core kinetochore receptor of RZZ. Indeed, the authors show that Knl1 depletion on its own already significantly decreases kinetochore levels of RZZ in HeLa cells, which agrees with prior studies in this cell line. However, prior studies in RPE1 cells reported little or no effect of Knl1 depletion on RZZ recruitment to kinetochores (Silió et al., 2015; Rodriguez-Rodriguez et al., 2018), which challenges the notion that Knl1 is the core receptor for RZZ in this diploid cell line. The authors have the perfect tools to clarify this important point using a similar strategy as for HeLa cells, i.e deplete Knl1 by RNAi (as done by Silió et al., 2015) in the RPE-1 CENP-E AID background and

electroporate in the R(EE)ZZ mutant. If Knl1 also acts as the core receptor for RZZ in RPE1 cells, RZZ should disappear from kinetochores in this condition.

We are grateful to the reviewer for this insightful suggestion. Following a recommendation by reviewer 2, further discussed with the Editor and approved by reviewer 1, we have now removed the part of the manuscript dealing with the identification of the additional kinetochore receptor of the RZZ complex. We will try this experiment for inclusion in our future work.

- Figure 2 - Supplement 1: Readers may not readily understand the difference between RZZS filaments (E) and mini-circles (F). As far as this reviewer understands, RZZS filaments are generated by mCherry-ROD/Zw10/Zwlich/Spindly, whereas mini-circles form when Spindly is additionally tagged with mCherry. If this is so, the label for RZZS in (E) needs to be changed.

We thank the reviewer for identifying this error in labelling, which we now have corrected in a revised version of the figure.

Also, why is EGFP alone recruited to mini-circles in (F)?

We apologize for the confusion. This resulted from choosing a suboptimal ROI for scaling, which caused its inadvertent, severe skewing in the GFP channel. We have now rescaled the images to provide a faithful representation of the GFP density in the negative control.

- Since different cell lines are used throughout the study, it would be helpful to indicate the specific cell line used for each experiment in the figure legends (this currently not the case for all legends).

We thank the reviewer for raising this point. We have now added the cell line information whenever missing (it was mostly for the "default" HeLa cell line).

Referee #2

The manuscript from Cmentowski et al. reports a number of interesting and important observations on motor proteins in the fibrous corona of the kinetochore in human cells. This effort continues a long-standing effort from the Musacchio group to reconstitute and understand the molecular interactions underlying formation and function of distinct sub-domains of the human kinetochore. Overall, the work is executed at a technically impressive level and the manuscript written with scholarly depth to place the findings in the context of the literature. While I am very supportive of the study, I did find it difficult to navigate the many different threads and a lack of differentiation between rigorously established findings and more speculative views on questions that will require future work to elucidate. The work also does not directly investigate the functional significance of the newly defined interactions beyond kinetochore localization analysis in nocodazole (note that the authors ability to rescue by electroporation with high quality recombinant proteins/complexes makes the localization analysis exceptionally rigorous - this is a strong point of the paper). Given the scope and rigor of work presented, the authors do not need to conduct any further functional analysis but to motivate others to take on this challenge it is important that the major findings be presented in a manner that is accessible and impactful in guiding the field.

We are grateful to the reviewer for this positive assessment of our work and for many helpful suggestions for streamlining the presentation

Major comments:

The manuscript presents 3 clear findings and the comments below are focused on each finding along with requests for clarification and suggestions on presentation:

i) The C-terminus of CENP-E contains independent sites for interaction with BubR1 (known from previous work) and RZZS; the BubR1 interaction is important for retention of CENP-E at aligned chromosomes and the RZZS interaction is important for CENP-E corona localization. Disrupting the RZZS interaction or both interactions negates the dominant-negative effect of over-expressing a C-terminal CENP-E fragment on chromosome alignment. These data shown in Figs 1 & 2 and associated supplemental figures are outstanding.

We thank the reviewers for these words of appreciation

With respect to the RZZS interaction, which is the major new finding here, have the authors tested direct interaction with the C-terminal CENP-E fragment using their gel filtration approach? Fig. 1H & I and the supplement presents TIRF-based analysis of interaction of the CENP-E C-terminal fragment with RZZS filaments and RZZS minicircles but not gel filtration (likely has been attempted and, even if negative, should be commented on).

We have indeed tried to detect an interaction in SEC experiments, but the results were inconclusive, not least because the elongation of the binding partners appeared to cause very small changes in the elution volumes of the individual species, so that it was difficult to make the call. We now indicate this at the end of the first section of Results: "We also used SEC as an alternative methodology to assess the CENP-E-RZZS interaction *in vitro*, but the outcome was inconclusive, as the extended conformation of the isolated binding partners caused them to elute, in isolation or as putative complex, at essentially identical volumes despite the different molecular mass (V.C. & A.M., unpublished results)."

The authors should emphasize the new interaction they identify (CENP-E with RZZS) by potentially moving their mutant design disrupting this interaction to the primary figures (the structural model in Fig. S1D is very compelling and should be a primary figure panel).

We think the reviewer refers to the AlphaFold2 structural model in Figure S2D. We have now rationalized the presentation of Figure 2 as clarified in a single response to the next point raised by the reviewer, which has also to do with the same figure.

Their one piece of functional analysis also strongly suggests that the RZZS-bound pool of CENP-E in the corona is important for chromosome alignment (comparing Fig. 2A to Fig. 2I, RZZS-Mut is only slightly better than BubR1-Mut in the localization analysis but significantly worse than BubR1-mut in the chromosome alignment analysis; this point is not coming through in part because these two figure panels are split apart - they should be presented adjacent to each other).

This is a good point and we have now reshuffled panels in figure 2 accordingly.

In addition, the FL CENP-E appears less sensitive to individual interaction perturbations than the C-terminal fragment (comparing Fig. 2A,B to Fig. 2G,H) - this should be commented on.

We have added a comment on this point: "The mutations seem to have a more penetrant effect in the context of EGFP-CENP-E^{2070C} than in the context of EGFP-CENP-E^{FL}, possibly because full-length CENP-E has another low-affinity kinetochore binding site that is deleted in CENP-E^{2070C} (compare quantifications in Figure 2B, E). This hypothetical site, however, is unable to

promote CENP-E localization when the BUBR1- and RZZS binding sites of CENP-E are mutated at the same time."

ii) Mps1 inhibition affects corona assembly (shown previously) but only modestly affects RZZS kinetochore localization; however, combining Mps1 inhibition with CENP-E depletion (which on its own has no significant effect on RZZS localization) leads to loss of RZZS from kinetochores. This is an important finding that is completely unexpected. The data are strong and conclusions are well supported. One prediction, that would help link these results to the first part of the manuscript, would be to analyze BubR1mut and RZZSmut CENP-E with Mps1 inhibition. Would both synergize with Mps1 inhibition in affecting RZZS localization? The authors do present data on the 2366C fragment of CENP-E (which lacks the BubR1 interaction site) but directly testing the mutants defined in the first part would help connect these two parts of the manuscript together.

We thank the reviewer for suggesting this experiment. We have now performed this experiment and included it as Figure EV3. The results demonstrate that either mutation, when combined with Reversine treatment and CENP-E depletion, affects the localization of RZZ to the kinetochore.

The figure would also be easier to interpret if the single Mps1 inhibition, shown in the supplement, was featured in the primary figure.

We appreciated the point raised by the reviewer, but also note that Figure 3 is already very packed. We have opted to display the experiment showing the single Reversine treatment as Figure EV2 (panels A-D). However, we have streamlined the presentation considerably and we feel that it is now much easier to read.

The authors next focus on dynein-dynactin localization (point iii below) but then follow with a section on how mimicking Mps1 phosphorylation of RZZ (specifically of ROD) bypasses the CENP-E & Mps1 activity requirement. It would be best if this section was moved up and immediately followed the rest of point ii - they are logically connected.

We agree with the reviewer, and note that this makes even more sense in view of the removal of the section on the elusive RZZS receptor. Therefore, we have swapped (former) Figures 4 and 5 and their description in the main text.

One conclusion from this section is that corona assembly does not require CENP-E or Mps1 activity if the two sites in Rod are phosphomimetic, highlighting the existence of a corona "receptor" that remains to be defined.

We concur that this is the main conclusion from this intermediate section

iii) CENP-E is important for dynein-dynactin recruitment to the corona. Despite over 3 decades of work on these two motors localized to kinetochores, it is remarkable that this relationship between them was missed (at least I was unable to find a simple deplete & localize experiment similar to what is reported here in prior literature). Note that Thrower et al. in the mid-90s had purified a minus end-directed motor activity associated with CENP-E but concluded that this activity was not dynein (PMID: 7889940); in hindsight, it seems likely that the activity may in fact be dynein (motility rate observed was consistent with that), which could be revisited with current mass spec and other approaches.

Thank you for pointing us to this reference, which we had missed and that we now introduce at the beginning of Discussion. We have also included another important reference that we had inadvertently omitted in our original manuscript (Cheerambatur et al. 2013).

The authors suggest that mutating the RZZS interface of CENP-E compromises its ability to recruit dynein-dynactin - however, as the RZZS mutant itself localizes worse than the WT 2070C fragment of CENP-E (Fig. 2A), this conclusion should be re-assessed after normalizing for the difference between WT and RZZS-Mut localization

We thank the reviewer for raising this very important point, that we had not considered with the required attention in our original submission. As this point related to the penultimate point here below, we respond both points together.

They also show, based on prior work, that residual dynein observed is likely being recruited by CENP-F. The authors test Spindly mutants that relieve its auto-inhibition and conclude that CENP-E does not act by relieving Spindly auto-inhibition (acknowledging that the mutants may not be "fully open"). Thus, the precise molecular reason for why CENP-E is needed to recruit dynein-dynactin remains unclear.

Indeed, our results do not identify the precise molecular basis of the role of CENP-E in DD recruitment

Once again, comparing WT, BubR1-mut and RZZS-mut CENP-E 2070C fragments for dynein-dynactin recruitment (normalizing for their own localization) may help tie the manuscript together - this is a suggestion rather than a requirement for revision and left to the authors' discretion.

This is an important consideration that we had missed in our original paper. We have now added a new Figure 5 – Supplement 1 where we provide quantifications normalized to the WT and RZZS mutant of CENP-E$^{2070\text{-}C}$ (the relevant comparison here). In one panel, we present the best-fit slopes for each normalized p150/EGFP data points in our data set, showing that the slope is steeper for WT than RZZS. In the other panel, we show the mean values of the p150/EGFP ratios. Both analyses are consistent with the tenet that the RZZS binding site of CENP-E is important for robust DD recruitment to kinetochores.

My strong recommendation to the authors is to end the manuscript at this point. The subsequent analysis, which does not lead to similarly strong conclusions, only serves to add complexity, dilute the impact of the findings above, and has caveats (all of which are properly acknowledged). These latter points are best saved for future manuscripts and leaving them out will in fact enhance the impact of the advances made by the authors. By taking some data/schematics from the supplement and integrating them into the manuscript structure, the authors will have presented an important and accessible contribution to addressing the relationship between the two key motor proteins at the kinetochore. A related suggestion would be for them to present a model figure that highlights the 3 key advances above and leave more complex schematics aimed at integration for a future review.

This is a very good point that connects with concerns raised by reviewer 1. After discussing this idea with the Editor and receiving approval, we now resubmit a revised version where the manuscript ends at the point suggested by the reviewer, and where we defer to further analyses for the identification of the still elusive RZZ receptor in the kinetochore.

Referee #3:

In this paper, Cmentowski and colleagues present a characterization of a molecular interaction network involving multiple components of the kinetochore corona, detailing both its assembly and its expansion mechanisms. Corona is a transient structure that assembles on the outer kinetochore during early mitosis and is essential for timely and error-free chromosome bi-orientation, alignment, and microtubule nucleation from kinetochores. Corona also serves as a platform for timely activation and deactivation of the spindle assembly checkpoint. Although highly studied in both functional and structural sense during the last decade, the understanding of detailed interaction networks that are responsible for corona assembly, maintenance, and expansion is still hampered by the large number of components involved and their complex molecular and regulatory interconnectivity, including, among others, the RZZ complex and Spindly (RZZS), CENP-E, CENP-F, Dynein-Dynactin (DD), Mad1:Mad2 complex and numerous kinases and phosphatases of which MPS1 kinase seems to play a central role in corona assembly. To battle with this complexity of interactions within the corona, the authors used a large set of state-of-the-art separation mutants of multiple corona components, most of which were developed in this paper, together with classic depletion approaches by RNA interference and auxin-inducible protein degradation. First, the authors show that CENP-E, a motor protein that resides in the kinetochore corona and mediates chromosome alignment, interacts not only with BUBR1, as previously shown, but also with the RZZ complex, and does so by using distinct regions. The authors were able to determine exact residues responsible for the interaction of CENP-E with both the BUBR1 and RZZ complex and further mutate these regions to establish a specific separation of CENP-E function mutants. They showed that mutants are localized to different parts of the kinetochore substructure, the outer kinetochore for BUBR1, and the corona for the RZZ-binding region. Thus, the authors showed that these distinct regions independently promote the recruitment of CENP-E to the kinetochore. The authors also show that after the establishment of biorientation and the loss of the corona, BUBR1 is the only receptor that mediates CENP-E localization on the kinetochore. Furthermore, the authors show that CENP-E is involved in the recruitment of RZZS to the kinetochore. However, CENP-E is not required for the recruitment of RZZs to the corona when the structure is preestablished, but is instead essential for the recruitment of RZZs when the corona assembly is perturbed by inhibition of MPS1. Furthermore, the authors showed that CENP-E contributes to the accumulation of Dynein-Dynactin to the kinetochore, presumably independently of the well-characterized DD kinetochore adaptor Spindly, together with the corona protein CENP-F. The authors further confirm that the corona can assemble without CENP-E by exploiting RZZ phosphomimetic mutants that mimic MPS1 phosphorylation of the ROD protein. By using additional separation of function mutants of the RZZ complex and Spindly depletion, both of which block corona expansion independently of MPS1, the authors were able to show that MPS1 is involved in the recruitment of RZZs to the kinetochore independently of its function in corona expansion, suggesting an additional kinetochore receptor for RZZs in addition to CENP-E. Finally, the authors were able to show through the use of RZZ phosphomimetic mutants that the kinetochore protein KNL1, either directly or indirectly, plays an important role in the recruitment of the RZZ complex, with a minor role of NDC80 and BUB1.

I think this is a highly valuable study that explores and substantially advances our knowledge on one of the most fundamental aspects of kinetochore function during early mitosis, namely corona assembly and shedding. I believe that the study will be highly relevant for researchers studying signaling during cell division, mechanisms of chromosome alignment, molecular motors, and the structure of the kinetochore. What I would like to emphasize as a strong feature of this paper is its thorough use of various separation-of-function mutants to pinpoint the molecular determinants of the localization of various interdependent components within this highly complex structure. In

that regard, it is my opinion that the approach presented here could be used as a role model of how similarly complex interaction networks within cells could be studied. The manuscript is written in a clear style, the figures are presented in such a detailed way, the experimental logic behind each hypothesis is elaborated, and the control experiments were carried out in such a rigorous way that I can only congratulate the authors for their work on this 'dauntingly complex process.' Therefore, it is my opinion that the manuscript is acceptable for publication in the EMBO Journal without the need for a major revision. I have, however, few minor comments that I depict below, in which I suggest the authors to elaborate more on certain points, or reanalyze some of the data to make some points within the text clearer.

We are grateful to the reviewer for these encouraging comments

Minor points:

1) The title of the manuscript in its current form is a little bit vague, and I would suggest trying something more specific as the title. This is due to the use of the "integration of microtubule motors of opposite polarity", which seems a bit premature to state here, in my opinion, since this scaffold would presumably influence the function of these proteins in chromosome biorientation, alignment, and SAC satisfaction, and for testing that the manuscript is currently lacking more direct functional approaches.

We have considered the reviewer's suggestion. While we quite liked the original title, we also appreciate that the emphasis on a "A mechanism…" might have been excessive. We have now revised the title as follows: "RZZ-Spindly and CENP-E form an integrated platform for robust recruitment of Dynein to the kinetochore corona"

2) It would be useful for the authors to quantify the level of colocalization of the CENP-E mutants BUBR1Mut and RZZSMut with Zwilch and CENP-C in the experiment presented in Figure 2A-C. This would substantiate the notion that the BUBR1 region is mainly mediated by the kinetochore localization of CENP-E, presumably implying a strong colocalization with CENP-C and less strong with Zwilch, and exactly the opposite for the RZZS region decorating more the corona region. See also point 7.

We address this important point more thoroughly in the context of point 7.

3) It is puzzling to me that the normalized signal intensities of various kinetochore components across multiple different experiments in control conditions seem to be very dispersed and in certain rare cases close to or even below the zero value, matching the low or absent signal when components were depleted or removed by other means. Is this due to low values in certain kinetochores or do whole cells sometimes have low values? I would comment on possible reasons in the figure caption or in Methods, as this seems to be a general phenomenon across different measurements.

Low values – including negative values – can be due background subtraction if the latter is high or the quantified signal is low. These negative values have no physical meaning, but we consider it more correct to retain them than to exclude them. The dispersion of values above the mean or median may be caused by overlapping ROIs, which are not completely infrequent given the close spacing of kinetochores/coronas. We now explain this in the Methods section.

4) The experiment that worried me the most is the one presented in Figure 2I. I did not fully understand how the authors came to the conclusion that simultaneous interaction with RZZS and BUBR1 stabilizes CENP-E from the data presented on Figure2I.

We have reconsidered our interpretation after reading this comment by the reviewer, and agree that the objection is valid and that the experiment does not provide the strongest basis for reaching such conclusions. We have therefore revised the main text and removed the sentence "These observations suggest that simultaneous interactions with RZZS and BUBR1 stabilize CENP-E."

The authors should first statistically compare the effects of various mutants on chromosome alignment presented now in Figure 2I.

We have followed the reviewer's suggestion and included a statistical analysis of these data and added SEM and results of t-test.

Second, I would recommend at least speculating on what the possible basis would be for the differential ability of mutants to displace endogenous CENP-E, especially since CENP-E2070C and its BUBR1Mut look very similar in terms of their effects on chromosome alignment, contrary to other two mutants.

Thank you for this suggestion. We now write "These results suggest that integration in the corona (possible for the wild type and BUBR1^{Mut} constructs) is important for the dominant-negative effects of CENP-E^{2070C} on chromosome alignment, also implying that integration in the corona is crucial for the chromosome alignment role of CENP-E"

Furthermore, the authors should speculate which of these mutants would be able to restore chromosome alignment in the case endogenous CENP-E is depleted and specific FL mutants of CENP-E (BUBR1Mut and RZZSMut) are expressed with functional motor domain.

Please see our answer to the previous point, which is related to this.

5) The authors should define the abbreviation 'CENP-EAID' when using it for the first time on page 9, similarly as the authors have done for other abbreviations in the manuscript. Currently it is first mentioned in the last paragraph on page 9, although the method was explained already in the preceding paragraph.

We have now rationalized the presentation of this abbreviation in a way that we think responds to the reviewer's concern.

6) I was a little puzzled why the authors studied in such detail the possible role of CENP-E in relieving Spindly autoinhibition and DD recruitment (Figures 4E-M), when at the end of the previous paragraph the authors presented an experiment that implied that "CENP-E does not contribute to DD recruitment by controlling Spindly levels". Although I understand the logic behind this, for the ease of reading I would suggest better linking of two parts, thus explaining the logic behind auto-inhibition path more clearly.

This has now become Figure 5E-M. We will not repeat the logic of these experiments here, as it is clear to the reviewer. We have nevertheless slightly rephrased this section to improve clarity. It is true that these results do not conclusively shed light on the mechanism through which CENP-E recruits DD. Yet, we felt it was important to test the hypothesis that CENP-E is possibly only

required to “open” Spindly. Our results imply that this is not the case, and that CENP-E does something more fundamental to promote the interaction with DD.

7) On page 16, in the Discussion part of the manuscript, the authors state that "The interaction with BUBR1 is dispensable for kinetochore recruitment of CENP-E in prometaphase, but CENP-E is clearly identified in corona, but becomes essential for kinetochore recruitment of CENP-E after corona shedding...". I don't understand the logic behind this conclusion. I mean if experimentally BUBR1 is depleted, will CENP-E be present at the outer kinetochore, at the place where it colocalizes with CENP-C but not with Zwilch and corona? In other words, how much CENP-E colocalizes with CENP-C in the absence of BUBR1 or in RZZSMut, where the interaction is presumably mediated only by BUBR1? This is related to point 2, and to the theme of two pools of CENP-E the authors discussed in Discussion part of the Manuscript.

We note that Figure 1D already provides a partial answer to the reviewer’s question. When we normalize signals to CENP-C, we only calculate the levels of CENP-E in the CENP-C mask. Therefore, we can conclude that the depletion of BUBR1 leads to a reduction of CENP-E in the CENP-C mask of approximately 30 %, i.e. the levels go from 1 to 0.71. The next condition, Zwilch RNAi, shows the residual levels of CENP-E, and also in this case in the CENP-C mask. Here, we see an even stronger reduction (to 0.5). Our interpretation of this is that the kinetochore (CENP-C) and corona signals of CENP-E overlap strongly, at least at the resolution of our images.

To answer the reviewer’s question in more detail, we developed a pipeline to carry out simple algebraic calculations on masks obtained from kinetochore and corona signals for the various antigens. These calculations were carried out on maximum intensity projections and were satisfactory for round kinetochore signals, but proved to be entirely unreliable for the coronas. We suspect that this analysis will require building a more sophisticated data collection and analysis strategy focusing on 3D reconstructions. While we see this as a priority for our future work, we also consider it beyond the scope of the present paper.

8) On page 18 when stating "While until now corona has been primarily viewed as a platform for the coordination of Dynein motility and spindle assembly checkpoint activity," I would suggest citing a review paper.

We followed the reviewer’s recommendation

Dr. Andrea Musacchio
Max Planck Institute of Molecular Physiology
Mechanistic Cell Biology
Otto Hahn Strasse 11
Dortmund 44227
Germany

24th Oct 2023

Re: EMBOJ-2023-114838R
RZZ-Spindly and CENP-E form an integrated platform to recruit Dynein to the kinetochore corona

Dear Andrea,

Thank you for submitting your revised manuscript to The EMBO Journal. One of the original referees has once more looked into it and assessed your responses (see below), and was fully satisfied with your revisions. We are therefore happy to accept the study for publication, following incorporation of the following few editorial points:

- First of all, we still need you to complete the Source Data (SD) checklist sent to you by my colleague Hannah Sonntag in her earlier message (the file you had returned was unfortunately blank - I am attaching it here once more). In it, you need to fill in the new panel number for each of the listed panels of the original submission, and whether SD has been provided (by uploading to our system or to BioStudies). The blue boxes at the bottom should be used to explain if in certain cases no Source Data was provided (e.g. because a figure was taken out in the final version), and to indicate BioStudies accession numbers and URLs in cases where the respective SD has only been externally deposited but not uploaded to our system.

- Related to this, the Biostudies Accession ID currently indicated in the Data Availability section of the manuscript appears to point to an unrelated work by another group - please check and correct.

- Also related to figures and original data: our usual pre-acceptance image checks indicated that several microscopy images replicated in different figures have not (or wrongly) been indicated/justified in the respective figure legends:
Figure legend EV2H call out to re-use of micrographs should be to Figure 3D, not Figure 3B?
Re-display of several (control) images from EV2E in Appendix Fig S2F needs to be indicated in both respective figure legends
In Figure EV3B, the DAPI/RZZSMut micrograph appears to have been mistakenly duplicated from Appendix Figure S2B - as it does not correspond to the other micrographs in that row; please double-check and clarify

- On the title page, please reduce the number of keyword terms to 5, ideally choosing general terms/concepts.

- Please add the section heading "Abstract" and "Introduction" to the respective sections

- Please rename the Declaration of interests section into Disclosure And Competing Interests Statement as specified in our Guide to Authors - for details, see https://www.embopress.org/competing-interests

- As we are switching from a free-text author contribution statement towards a more formal statement based on Contributor Role Taxonomy (CRediT) terms, please remove the present Author Contribution section and instead specify each author's contribution(s) directly in the Author Information page of our submission system during upload of the final manuscript. See https://casrai.org/credit/ for more information.

- Please also double-check the updated email account giuseppe.ciossani@gmail.com, as our subsequent acknowledgement email seemed again to be undeliverable.

- Please remove the Appendix Figure List from the main manuscript text, this should only be present in the Appendix PDF itself. In the Appendix, it would be ideal to place each legend directly underneath the respective figure, as the Appendix will not be typeset/reformatted.

- Finally, please provide suggestions for a short 'blurb' text prefacing and summing up the conceptual aspect of the study in two sentences (max. 250 characters), followed by 3-5 one-sentence 'bullet points' with brief factual statements of key results of the paper; they will form the basis of an editor-written 'Synopsis' accompanying the online version of the article. Please also upload a synopsis image, which can be used as a "visual title" for the synopsis section of your paper. The image (maybe a much simplified and non-background-colored version of Figure 6A?) should be in PNG or JPG format, and please make sure that it remains in the modest dimensions of (exactly) 550 pixels wide and 300-600 pixels high.

I am therefore returning the manuscript to you for a final round of minor revision, to allow you to make these adjustments and upload all modified files. Once we will have received them, we should be ready to swiftly proceed with formal acceptance and production of the manuscript.

With kind regards,

Hartmut

Hartmut Vodermaier, PhD
Senior Editor, The EMBO Journal
h.vodermaier@embojournal.org

*** PLEASE NOTE: All revised manuscript are subject to initial checks for completeness and adherence to our formatting guidelines. Revisions may be returned to the authors and delayed in their editorial re-evaluation if they fail to comply to the following requirements (see also our Guide to Authors for further information):

1) Every manuscript requires a Data Availability section (even if only stating that no deposited datasets are included). Primary datasets or computer code produced in the current study have to be deposited in appropriate public repositories prior to resubmission, and reviewer access details provided in case that public access is not yet allowed. Further information: embopress.org/page/journal/14602075/authorguide#dataavailability

2) Each figure legend must specify
- size of the scale bars that are mandatory for all micrograph panels
- the statistical test used to generate error bars and P-values
- the type error bars (e.g., S.E.M., S.D.)
- the number (n) and nature (biological or technical replicate) of independent experiments underlying each data point
- Figures may not include error bars for experiments with n<3; scatter plots showing individual data points should be used instead.

3) Revised manuscript text (including main tables, and figure legends for main and EV figures) has to be submitted as editable text file (e.g., .docx format). We encourage highlighting of changes (e.g., via text color) for the referees' reference.

4) Each main and each Expanded View (EV) figure should be uploaded as individual production-quality files (preferably in .eps, .tif, .jpg formats). For suggestions on figure preparation/layout, please refer to our Figure Preparation Guidelines: http://bit.ly/EMBOPressFigurePreparationGuideline

5) Point-by-point response letters should include the original referee comments in full together with your detailed responses to them (and to specific editor requests if applicable), and also be uploaded as editable (e.g., .docx) text files.

6) Please complete our Author Checklist, and make sure that information entered into the checklist is also reflected in the manuscript; the checklist will be available to readers as part of the Review Process File. A download link is found at the top of our Guide to Authors: embopress.org/page/journal/14602075/authorguide

7) All authors listed as (co-)corresponding need to deposit, in their respective author profiles in our submission system, a unique ORCiD identifier linked to their name. Please see our Guide to Authors for detailed instructions.

8) Please note that supplementary information at EMBO Press has been superseded by the 'Expanded View' for inclusion of additional figures, tables, movies or datasets; with up to five EV Figures being typeset and directly accessible in the HTML version of the article. For details and guidance, please refer to: embopress.org/page/journal/14602075/authorguide#expandedview

9) Digital image enhancement is acceptable practice, as long as it accurately represents the original data and conforms to community standards. If a figure has been subjected to significant electronic manipulation, this must be clearly noted in the figure legend and/or the 'Materials and Methods' section. The editors reserve the right to request original versions of figures and the original images that were used to assemble the figure. Finally, we generally encourage uploading of numerical as well as gel/blot image source data; for details see: embopress.org/page/journal/14602075/authorguide#sourcedata

At EMBO Press, we ask authors to provide source data for the main manuscript figures. Our source data coordinator will contact you to discuss which figure panels we would need source data for and will also provide you with helpful tips on how to upload and organize the files.

Further information is available in our Guide For Authors:
https://www.embopress.org/page/journal/14602075/authorguide

In the interest of ensuring the conceptual advance provided by the work, we recommend submitting a revision within 3 months (22nd Jan 2024). Please discuss the revision progress ahead of this time with the editor if you require more time to complete the revisions. Use the link below to submit your revision:

Link Not Available

Referee #1:

The authors have addressed all of my initial concerns.

Max-Planck-Institute for molecular Physiology
Department for Mechanistic Cell Biology

- First of all, we still need you to complete the Source Data (SD) checklist sent to you by my colleague Hannah Sonntag in her earlier message (the file you had returned was unfortunately blank - I am attaching it here once more). In it, you need to fill in the new panel number for each of the listed panels of the original submission, and whether SD has been provided (by uploading to our system or to BioStudies). The blue boxes at the bottom should be used to explain if in certain cases no Source Data was provided (e.g. because a figure was taken out in the final version), and to indicate BioStudies accession numbers and URLs in cases where the respective SD has only been externally deposited but not uploaded to our system.

We have uploaded the compiled Source Data checklist and indicated differences with the previous version of the manuscript.

- Related to this, the Biostudies Accession ID currently indicated in the Data Availability section of the manuscript appears to point to an unrelated work by another group - please check and correct.

The link was incorrect due to the loss of a final ‘1’ that has been added back. The link works now

- Also related to figures and original data: our usual pre-acceptance image checks indicated that several microscopy images replicated in different figures have not (or wrongly) been indicated/justified in the respective figure legends:

We apologize for these omissions, which had escaped our attention. We are very grateful for these pre-publication check, which helps us identify involuntary omissions.

Figure legend EV2H call out to re-use of micrographs should be to Figure 3D, not Figure 3B?

Indeed. Apologies for forgetting to report this duplication. We now write: “Representative images showing the localization of MAD1 in RPE-1 CENP-EAID_3xFLAG cells treated as shown in Figure 3B. This mount is part of a larger experiment in which Zwilch was also visualized (in Figure 3D; omitted here). Therefore, the images in the CENP-C and DAPI channels are duplicates of those shown in Figure 3D, where MAD1 was instead omitted.” The reference to Figure 3B is only meant to indicate the protocol used for these experiments.

We have included a similar call within the legend to Figure 3D: “The mount is part of a larger experiment in which MAD1 was also visualized (displayed in Figure EV2H; omitted here). Therefore, the images in the CENP-C and DAPI channels are duplicates of those shown in Figure EV2H (where Zwilch was omitted).”

Re-display of several (control) images from EV2E in Appendix Fig S2F needs to be indicated in both respective figure legends

Again, sorry for forgetting to report this. We now indicate the existence of this duplication in both figure legends. We also clarify that this control experiment with the WT construct contained DMSO, as it was part of a larger experiment where we added or omitted Reversine (dissolved in DMSO, and therefore DMSO is added to our control and to all other samples not treated with Reversine). We now clarify this point in the legend to Appendix Fig S2F.

In Figure EV3B, the DAPI/RZZSMut micrograph appears to have been mistakenly

duplicated from Appendix Figure S2B - as it does not correspond to the other micrographs in that row; please double-check and clarify.

This was a plain mistake that occurred when importing the panels in Illustrator. We have now corrected this mistake (and also added a missing "EGFP") and have uploaded revised figures.

- On the title page, please reduce the number of keyword terms to 5, ideally choosing general terms/concepts.

Done

- Please add the section heading "Abstract" and "Introduction" to the respective sections

Done

- Please rename the Declaration of interests section into Disclosure And Competing Interests Statement as specified in our Guide to Authors - for details, see https://www.embopress.org/competing-interests

Done

- As we are switching from a free-text author contribution statement towards a more formal statement based on Contributor Role Taxonomy (CRediT) terms, please remove the present Author Contribution section and instead specify each author's contribution(s) directly in the Author Information page of our submission system during upload of the final manuscript. See https://casrai.org/credit/ for more information.

Done

- Please also double-check the updated email account giuseppe.ciossani@gmail.com, as our subsequent acknowledgement email seemed again to be undeliverable.

Apologies for this. The correct email is now in place

- Please remove the Appendix Figure List from the main manuscript text, this should only be present in the Appendix PDF itself. In the Appendix, it would be ideal to place each legend directly underneath the respective figure, as the Appendix will not be typeset/reformatted.

Done

- Finally, please provide suggestions for a short 'blurb' text prefacing and summing up the conceptual aspect of the study in two sentences (max. 250 characters), followed by 3-5 one-sentence 'bullet points' with brief factual statements of key results of the paper; they will form the basis of an editor-written 'Synopsis' accompanying the online version of the article. Please also upload a synopsis image, which can be used as a "visual title" for the synopsis section of your paper. The image (maybe a much simplified and non-background-colored version of Figure 6A?) should be in PNG or JPG format, and please make sure that it remains in the modest dimensions of (exactly) 550 pixels wide and 300-600 pixels high.

Done

2nd Revision - Editorial Decision 26th Oct 2023

Dr. Andrea Musacchio
Max Planck Institute of Molecular Physiology
Mechanistic Cell Biology
Otto Hahn Strasse 11
Dortmund 44227
Germany

26th Oct 2023

Re: EMBOJ-2023-114838R1
RZZ-Spindly and CENP-E form an integrated platform to recruit Dynein to the kinetochore corona

Dear Andrea,

Thank you for submitting your final revised manuscript for our consideration. I am pleased to inform you that we have now accepted it for publication in The EMBO Journal.

Your article will be processed for publication in The EMBO Journal by EMBO Press and Wiley, who will contact you with further information regarding production/publication procedures and license requirements. You will also be provided with page proofs after copy-editing and typesetting of main manuscript and expanded view figure files.

Should you be planning a Press Release on your article, please get in contact with embojournal@wiley.com as early as possible, in order to coordinate publication and release dates.

Thank you again for this contribution to The EMBO Journal and congratulations on a successful publication! Please consider us again in the future for your most exciting work.

With kind regards,

Hartmut

Hartmut Vodermaier, PhD
Senior Editor, The EMBO Journal
h.vodermaier@embojournal.org

EMBO Press Author Checklist

Corresponding Author Name: Musacchio & Cmentowski	
Journal Submitted to: EMBO J	
Manuscript Number: EMBOJ-2023-114838	

USEFUL LINKS FOR COMPLETING THIS FORM
The EMBO Journal - Author Guidelines
EMBO Reports - Author Guidelines
Molecular Systems Biology - Author Guidelines
EMBO Molecular Medicine - Author Guidelines

Reporting Checklist for Life Science Articles (updated January
This checklist is adapted from Materials Design Analysis Reporting (MDAR) Checklist for Authors. MDAR establishes a minimum set of requirements in transparent reporting in the life sciences (see Statement of Task: 10.31222/osf.io/9sm4x). Please follow the journal's guidelines in preparing your
Please note that a copy of this checklist will be published alongside your article.

Abridged guidelines for figures
1. Data
The data shown in figures should satisfy the following conditions:
- the data were obtained and processed according to the field's best practice and are presented to reflect the results of the experiments in an accurate and unbiased manner.
- ideally, figure panels should include only measurements that are directly comparable to each other and obtained with the same assay.
- plots include clearly labeled error bars for independent experiments and sample sizes. Unless justified, error bars should not be shown for technical
- if n<5, the individual data points from each experiment should be plotted. Any statistical test employed should be justified.
- Source Data should be included to report the data underlying figures according to the guidelines set out in the authorship guidelines on Data

2. Captions
Each figure caption should contain the following information, for each panel where they are relevant:
- a specification of the experimental system investigated (eg cell line, species name).
- the assay(s) and method(s) used to carry out the reported observations and measurements.
- an explicit mention of the biological and chemical entity(ies) that are being measured.
- an explicit mention of the biological and chemical entity(ies) that are altered/varied/perturbed in a controlled manner.
- the exact sample size (n) for each experimental group/condition, given as a number, not a range;
- a description of the sample collection allowing the reader to understand whether the samples represent technical or biological replicates (including how many animals, litters, cultures, etc.).
- a statement of how many times the experiment shown was independently replicated in the laboratory.
- definitions of statistical methods and measures:
  - common tests, such as t-test (please specify whether paired vs. unpaired), simple χ2 tests, Wilcoxon and Mann-Whitney tests, can be unambiguously identified by name only, but more complex techniques should be described in the methods section;
  - are tests one-sided or two-sided?
  - are there adjustments for multiple comparisons?
  - exact statistical test results, e.g., P values = x but not P values < x;
  - definition of 'center values' as median or average;
  - definition of error bars as s.d. or s.e.m.

Please complete ALL of the questions below.
Select "Not Applicable" only when the requested information is not relevant for your study.

Materials

Newly Created Materials	Information included in the manuscript?	In which section is the information available? (Reagents and Tools Table, Materials and Methods, Figures, Data Availability Section)
New materials and reagents need to be available; do any restrictions apply?	Yes	Materials and Methods

Antibodies	Information included in the manuscript?	In which section is the information available? (Reagents and Tools Table, Materials and Methods, Figures, Data Availability Section)
For **antibodies** provide the following information:<br>- Commercial antibodies: RRID (if possible) or supplier name, catalogue number and or/clone number<br>- Non-commercial: RRID or citation	Yes	Materials and Methods

DNA and RNA sequences	Information included in the manuscript?	In which section is the information available? (Reagents and Tools Table, Materials and Methods, Figures, Data Availability Section)
**Short novel DNA or RNA including primers, probes**: provide the sequences.	Yes	Materials and Methods

Cell materials	Information included in the manuscript?	In which section is the information available? (Reagents and Tools Table, Materials and Methods, Figures, Data Availability Section)
**Cell lines:** Provide species information, strain. Provide accession number in repository **OR** supplier name, catalog number, clone number, and/**OR** RRID.	Yes	Materials and Methods
**Primary cultures:** Provide species, strain, sex of origin, genetic modification status.	Not Applicable	
Report if the cell lines were recently **authenticated** (e.g., by STR profiling) and tested for mycoplasma contamination.	Yes	Regular tests for Mycoplasma

Experimental animals	Information included in the manuscript?	In which section is the information available? (Reagents and Tools Table, Materials and Methods, Figures, Data Availability Section)
**Laboratory animals or Model organisms:** Provide species, strain, sex, age, genetic modification status. Provide accession number in repository **OR** supplier name, catalog number, clone number, **OR** RRID.	Not Applicable	
**Animal observed in or captured from the field:** Provide species, sex, and age where possible.	Not Applicable	
Please detail **housing and husbandry conditions**.	Not Applicable	

Plants and microbes	Information included in the manuscript?	In which section is the information available? (Reagents and Tools Table, Materials and Methods, Figures, Data Availability Section)
**Plants:** provide species and strain, ecotype and cultivar where relevant, unique accession number if available, and source (including location for collected wild specimens).	Not Applicable	
**Microbes:** provide species and strain, unique accession number if available, and source.	Not Applicable	

Human research participants	Information included in the manuscript?	In which section is the information available? (Reagents and Tools Table, Materials and Methods, Figures, Data Availability Section)
If collected and within the bounds of privacy constraints report on age, sex and gender or ethnicity for all study participants.	Not Applicable	

Core facilities	Information included in the manuscript?	In which section is the information available? (Reagents and Tools Table, Materials and Methods, Figures, Data Availability Section)
If your work benefited from core facilities, was their service mentioned in the acknowledgments section?	Not Applicable	

Design

Study protocol	Information included in the manuscript?	In which section is the information available? (Reagents and Tools Table, Materials and Methods, Figures, Data Availability Section)
If study protocol has been **pre-registered, provide DOI in the manuscript**. For clinical trials, provide the trial registration number **OR** cite DOI.	Not Applicable	
Report the **clinical trial registration number** (at ClinicalTrials.gov or equivalent), where applicable.	Not Applicable	

Laboratory protocol	Information included in the manuscript?	In which section is the information available? (Reagents and Tools Table, Materials and Methods, Figures, Data Availability Section)
Provide DOI OR other citation details if **external detailed step-by-step protocols** are available.	Not Applicable	

Experimental study design and statistics	Information included in the manuscript?	In which section is the information available? (Reagents and Tools Table, Materials and Methods, Figures, Data Availability Section)
Include a statement about **sample size** estimate even if no statistical methods were used.	Not Applicable	
Were any steps taken to minimize the effects of subjective bias when allocating animals/samples to treatment (e.g. **randomization procedure**)? If yes, have they been described?	Not Applicable	
Include a statement about **blinding** even if no blinding was done.	Not Applicable	
Describe **inclusion/exclusion criteria** if samples or animals were excluded from the analysis. Were the criteria pre-established?<br><br>If sample or data points were omitted from analysis, report if this was due to attrition or intentional exclusion and provide justification.	Not Applicable	
For every figure, are **statistical tests** justified as appropriate? Do the data meet the assumptions of the tests (e.g., normal distribution)? Describe any methods used to assess it. Is there an estimate of variation within each group of data? Is the variance similar between the groups that are being statistically compared?	Yes	Materials and Methods

Sample definition and in-laboratory replication	Information included in the manuscript?	In which section is the information available? (Reagents and Tools Table, Materials and Methods, Figures, Data Availability Section)
In the figure legends: state number of times the experiment was **replicated** in laboratory.	Yes	Figure Legends
In the figure legends: define whether data describe **technical or biological replicates**.	Yes	Figure Legends

Ethics

Ethics	Information included in the manuscript?	In which section is the information available? (Reagents and Tools Table, Materials and Methods, Figures, Data Availability Section)
Studies involving **human participants**: State details of **authority granting ethics approval** (IRB or equivalent committee(s), provide reference number for approval.	Not Applicable	
Studies involving **human participants**: Include a statement confirming that **informed consent** was obtained from all subjects and that the experiments conformed to the principles set out in the WMA Declaration of Helsinki and the Department of Health and Human Services Belmont Report.	Not Applicable	
Studies involving **human participants:** For publication of **patient photos**, include a statement confirming that consent to publish was obtained.	Not Applicable	
Studies involving experimental **animals**: State details of **authority granting ethics approval** (IRB or equivalent committee(s), provide reference number for approval. Include a statement of compliance with ethical regulations.	Not Applicable	
Studies involving **specimen and field samples:** State if relevant **permits** obtained, provide details of authority approving study; if none were required, explain why.	Not Applicable	

Dual Use Research of Concern (DURC)	Information included in the manuscript?	In which section is the information available? (Reagents and Tools Table, Materials and Methods, Figures, Data Availability Section)
Could your study fall under dual use research restrictions? Please check biosecurity documents and list of **select agents and toxins** (CDC): https://www.selectagents.gov/sat/list.htm	Not Applicable	
If you used a select agent, is the security level of the lab appropriate and reported in the manuscript?	Not Applicable	
If a study is subject to dual use research of concern regulations, is the name of the **authority granting approval and reference number** for the regulatory approval provided in the manuscript?	Not Applicable	

Reporting

The MDAR framework recommends adoption of discipline-specific guidelines, established and endorsed through community initiatives. Journals have their own policy about requiring specific guidelines and recommendations to complement MDAR.

Adherence to community standards	Information included in the manuscript?	In which section is the information available? (Reagents and Tools Table, Materials and Methods, Figures, Data Availability Section)
State if relevant guidelines or checklists (e.g., **ICMJE, MIBBI, ARRIVE, PRISMA**) have been followed or provided.	Not Applicable	
For **tumor marker prognostic studies,** we recommend that you follow the **REMARK** reporting guidelines (see link list at top right). See author guidelines, under 'Reporting Guidelines'. Please confirm you have followed these guidelines.	Not Applicable	
For **phase II and III randomized controlled trials**, please refer to the **CONSORT** flow diagram (see link list at top right) and submit the CONSORT checklist (see link list at top right) with your submission. See author guidelines, under 'Reporting Guidelines'. Please confirm you have submitted this list.	Not Applicable	

Data Availability

Data availability	Information included in the manuscript?	In which section is the information available? (Reagents and Tools Table, Materials and Methods, Figures, Data Availability Section)
Have **primary datasets** been deposited according to the journal's guidelines (see 'Data Deposition' section) and the respective accession numbers provided in the Data Availability Section?	Yes	Data Availability Section
Were **human clinical and genomic datasets** deposited in a public access-controlled repository in accordance to ethical obligations to the patients and to the applicable consent agreement?	Not Applicable	
Are **computational models** that are central and integral to a study available without restrictions in a machine-readable form? Were the relevant accession numbers or links provided?	Not Applicable	
If publicly available data were reused, provide the respective **data citations in the reference list**.	Not Applicable	
